# Antibiotic-Specific Genotype–Phenotype Concordance and Cross-Database Interoperability in Public *Escherichia coli/Shigella* WGS-AMR Metadata

**DOI:** 10.3390/ijms27167157

**Published:** 2026-08-10

**Authors:** Abdullateef Abdullah Alshehri

**Affiliations:** 1 Department of Clinical Laboratory Sciences, Faculty of Applied Medical Sciences, Najran University, P.O. Box 1988, Najran 61441, Saudi Arabia; aaalshehre@nu.edu.sa; Tel.: +966-17-5417524; 2 Department of Infection Prevention and Control, Najran University Hospital, Najran University, P.O. Box 1988, Najran 61441, Saudi Arabia; 3 Genomics and Genetics Unit, Health Research Center, Najran University, P.O. Box 1988, Najran 61441, Saudi Arabia

**Keywords:** antimicrobial resistance, whole-genome sequencing, genotype–phenotype concordance, *Escherichia coli*/*Shigella*, NCBI Pathogen Detection, BV-BRC, antimicrobial susceptibility testing, genomic surveillance

## Abstract

Public pathogen-genomics repositories are increasingly used for antimicrobial resistance (AMR) surveillance, yet the reliability and interoperability of database-derived genotype–phenotype inference remain incompletely characterized. This study evaluated antibiotic-specific concordance between exported AMR genotype annotations and antimicrobial susceptibility testing (AST) phenotypes in the NCBI Pathogen Detection metadata for the *Escherichia coli/Shigella* organism group and used BV-BRC to assess complementary phenotype-related coverage and cross-resource linkage. Frozen NCBI Pathogen Detection and BV-BRC exports were analyzed retrospectively. NCBI records were filtered for assembly accessions, AMR genotype annotations, and interpretable resistant or susceptible AST results. Prespecified antibiotic-specific mapping rules were applied. Performance metrics, including sensitivity, specificity, accuracy, balanced accuracy, positive and negative predictive values and F1-score, were calculated, and their 95% confidence intervals were estimated using 10,000 isolate-level bootstrap replicates. BV-BRC was evaluated descriptively for phenotype-related coverage, evidence type, and accession overlap. Among 539,918 NCBI records, 10,201 met prespecified criteria for assembly accession, AMR genotype annotation, and interpretable resistant/susceptible AST data. These records generated 56,141 genotype–phenotype comparisons across eight priority antibiotics. Concordance was high for tetracycline (accuracy, 98.4%; F1-score, 98.1%) and ceftriaxone (accuracy, 97.9%; F1-score, 94.6%), but lower for amoxicillin–clavulanic acid (accuracy, 73.6%; F1-score, 44.5%), indicating antibiotic-specific limits of genotype-field inference. Discordance was explicitly separated into 2524 genotype-positive/phenotype-susceptible and 923 phenotype-resistant observations with no mapped genomic evidence of resistance. BV-BRC contributed 21,298 taxonomy-strict *E. coli*/*Shigella* genomes and 4745 phenotype-linked identifiers. Although 15,214 BioSample and 10,121 assembly accessions were shared across exports, no analysis-ready overlap remained between the final NCBI genotype–AST and BV-BRC phenotype-linked subsets after eligibility filtering. Public WGS-AMR databases can support large-scale surveillance-oriented concordance analyses, but performance is antibiotic specific and depends on mapping rules, phenotype definitions, evidence provenance, and accession linkage. These estimates do not constitute clinical diagnostic validation and should not replace phenotypic AST. Because the analysis was conducted at the combined organism-group level, these estimates may mask species-, pathotype-, or lineage-specific resistance dynamics.

## 1. Introduction

Antimicrobial resistance (AMR) has emerged as one of the foremost threats to global health, with an estimated 1.27 million deaths attributable to bacterial AMR in 2019 and a projected increase in the global AMR burden through 2050 [1,2,3]. Antibiotic-resistant bacterial infections compromise the effective treatment of common infections and increase the risks associated with routine medical procedures. Genomic technologies such as whole-genome sequencing (WGS) complement conventional phenotypic workflows by enabling the detection of acquired resistance determinants and supporting in silico inference of antimicrobial susceptibility profiles [4,5,6]. Recognizing these advantages, clinical and public health laboratories have increasingly integrated WGS into pathogen surveillance, outbreak investigation, and antimicrobial resistance monitoring [3,7,8,9]. However, the value of WGS extends beyond genome generation and depends on standardized detection and interpretation of resistance determinants, transparent analytical workflows, curated reference databases, and interoperable genomic and phenotypic metadata [4,5,6,10,11].

Validation studies using AMRFinder-derived annotations and *Escherichia coli* isolate collections have demonstrated high genotype–phenotype concordance for several organism–antibiotic combinations, while also showing that predictive performance remains dependent on the antimicrobial agent, resistance mechanism, database content, analytical workflow, and interpretation framework [10,11,12,13,14]. Direct comparisons of phenotypic and WGS-derived AMR profiles in *Shigella* and enteroaggregative *E. coli* have similarly demonstrated the value of genomic resistance inference for public-health surveillance, while identifying discordance associated with incomplete or truncated determinants, promoter effects, allelic variation, gene expression, and antibiotic-specific interpretation [15,16]. Interlaboratory evaluations have further shown that identical WGS datasets may generate discordant AMR predictions when analytical pipelines, reference databases, parameter thresholds, and reporting rules differ [17]. Moreover, the close genomic relationship between *E. coli* and *Shigella* can complicate sequence-based taxonomic separation, emphasizing that analyses of combined public *E. coli*/*Shigella* metadata should be interpreted at the organism-group level unless reliable species-, pathotype-, or lineage-level classification is available [18,19]. Collectively, these findings support the use of public WGS resources for surveillance-oriented genotype–phenotype analysis, but they also emphasize that detection of a genomic resistance determinant should not automatically be interpreted as phenotypic resistance or clinical actionability. Reliable secondary analysis of public WGS-AMR data therefore requires transparent reporting of database provenance, phenotype definitions, accession linkage, analytical thresholds, genotype-to-antibiotic mapping rules, taxonomic resolution, and uncertainty.

Public WGS databases increasingly support AMR surveillance, outbreak investigation, source tracking, and risk assessment. However, their value depends not only on genome availability but also on the completeness, interpretability, and linkage of AMR genotype and AST phenotype metadata. Large-scale evaluation of genotype–phenotype concordance in public repositories can identify antibiotics with robust prediction performance, highlight lower-confidence drug categories, and clarify where genotype-only inference may be insufficient for clinical or surveillance interpretation.

Large public pathogen-genomics databases, including NCBI Pathogen Detection and BV-BRC, contain extensive bacterial genome metadata and AMR-related information [20,21]. These resources are valuable because they aggregate genome-linked AMR metadata across laboratories, countries, sources, and surveillance programs. However, they were not designed as perfectly harmonized clinical datasets [3,22,23]. Records may differ in assembly availability, phenotype completeness, antibiotic panels, susceptibility interpretation, accession linkage, and metadata structure. Therefore, public AMR database studies require explicit filtering rules and transparent reporting.

Genotype-based resistance prediction is generally more reliable for antibiotics for which resistance is primarily mediated by well-characterized genes or mutations [12,13,14,24,25,26]. However, resistance phenotypes may vary even among isolates with apparently similar resistance-gene profiles. Examples in Enterobacterales include mutations within β-lactamase promoters or transcriptional attenuator regions that alter gene expression, amplification or increased expression of chromosomal *ampC*; regulatory mutations affecting multidrug-efflux systems; and sequence changes or loss of outer-membrane porins that modify antimicrobial permeability. Porin alterations may further interact with ESBL, AmpC, or carbapenemase determinants to increase resistance despite otherwise similar acquired-gene profiles. Therefore, isolates with apparently comparable resistance-gene content may display different susceptibility phenotypes because of differences in gene expression, regulatory sequence, copy number, permeability, or epistatic interactions among mechanisms. Antibiotic-specific analysis is therefore essential because aggregate performance estimates can conceal these mechanism-dependent differences [8,12,27,28,29,30,31].

Discordant genotype–phenotype results are not merely errors; they can identify important biological, technical, and metadata-related limitations [12,25,32]. Genotype-positive/phenotype-susceptible records may reflect incomplete or non-expressed resistance determinants, uncertain allele effects, differences in gene regulation, or variation in phenotypic testing methods and interpretive breakpoints. Conversely, phenotype-resistant observations with no mapped genomic evidence of resistance may indicate uncharacterized mechanisms, regulatory changes, porin loss, efflux activation, incomplete resistance catalogs, or limitations in the exported genotype annotations. Previous studies have therefore emphasized the importance of reporting both directions of discordance separately rather than relying only on aggregate performance measures [12,25,32,33].

More broadly, studies using public genomic and AMR repositories have shown that reliable secondary analysis depends on the harmonization of phenotype definitions, evidence provenance, antibiotic nomenclature, accession identifiers, and genotype-to-antibiotic interpretation rules [10,20,21,22,23]. Distinguishing concordant-resistant, concordant-susceptible, genotype-positive/phenotype-susceptible, and phenotype-resistant/no-mapped-genomic-evidence outcomes provides a transparent framework for identifying antibiotic-specific strengths, sources of discordance, and limitations in data integration. Although WGS-based AMR concordance has been evaluated extensively in curated isolate collections, the reuse of large public metadata exports remains more challenging because genotype annotations, phenotype records, evidence types, and accession links may be incomplete, heterogeneous, or inconsistently structured across databases [20,23,34,35]. These limitations should be considered when interpreting public repository data for genomic AMR surveillance.

Although *E. coli* and *Shigella* are frequently organized within a combined organism group in public genomic repositories because of their close genomic relatedness, this database classification should not be interpreted as clinical, ecological, or evolutionary equivalence [18,19]. *Shigella* lineages are predominantly host-adapted enteric pathogens with distinct transmission patterns, pathogenic mechanisms, ecological constraints, and antimicrobial selection pressures, whereas *E. coli* encompasses commensal, intestinal pathogenic, and extraintestinal pathogenic lineages [19]. Consequently, analysis at the combined organism-group level may obscure species- or lineage-specific resistance dynamics, clonal expansions, and genotype–phenotype relationships. The findings of this study should therefore be interpreted as database-level estimates for the combined public *E. coli*/*Shigella* metadata group rather than as species- or lineage-specific performance estimates.

BV-BRC was included not as an external validation cohort but as a second public AMR metadata system through which database complementarity and interoperability could be evaluated. In the exports analyzed here, BV-BRC provided separately structured genome-linked phenotype-related records, evidence-method labels distinguishing Laboratory Method from Computational Method entries, antibiotic-coverage summaries, and independently organized genome and accession metadata. These features permitted assessment of whether phenotype-related information and shared genome identifiers from one public resource could be linked to the genotype–AST analytical subset derived from another.

Given these gaps, this study was designed as a retrospective computational genomic epidemiology analysis of public WGS-AMR metadata. Rather than independently benchmarking multiple AMR tools from raw assemblies, we evaluated genotype–phenotype concordance using AMR genotype annotations exported from NCBI Pathogen Detection and AST phenotype summaries available within the same public metadata source. BV-BRC was used as a complementary metadata resource to assess genome metadata structure, AMR phenotype availability, antibiotic coverage, and accession overlap. The primary objective was to quantify antibiotic-specific concordance between exported NCBI AMR genotype annotations and interpretable resistant or susceptible AST phenotypes. Secondary objectives were to characterize directional discordance, evaluate determinant-level patterns for the lowest-performing antibiotic, and assess phenotype coverage and accession interoperability between NCBI Pathogen Detection and BV-BRC.

## 2. Results

### 2.1. Dataset and Analytical Overview

**Figure 1:**
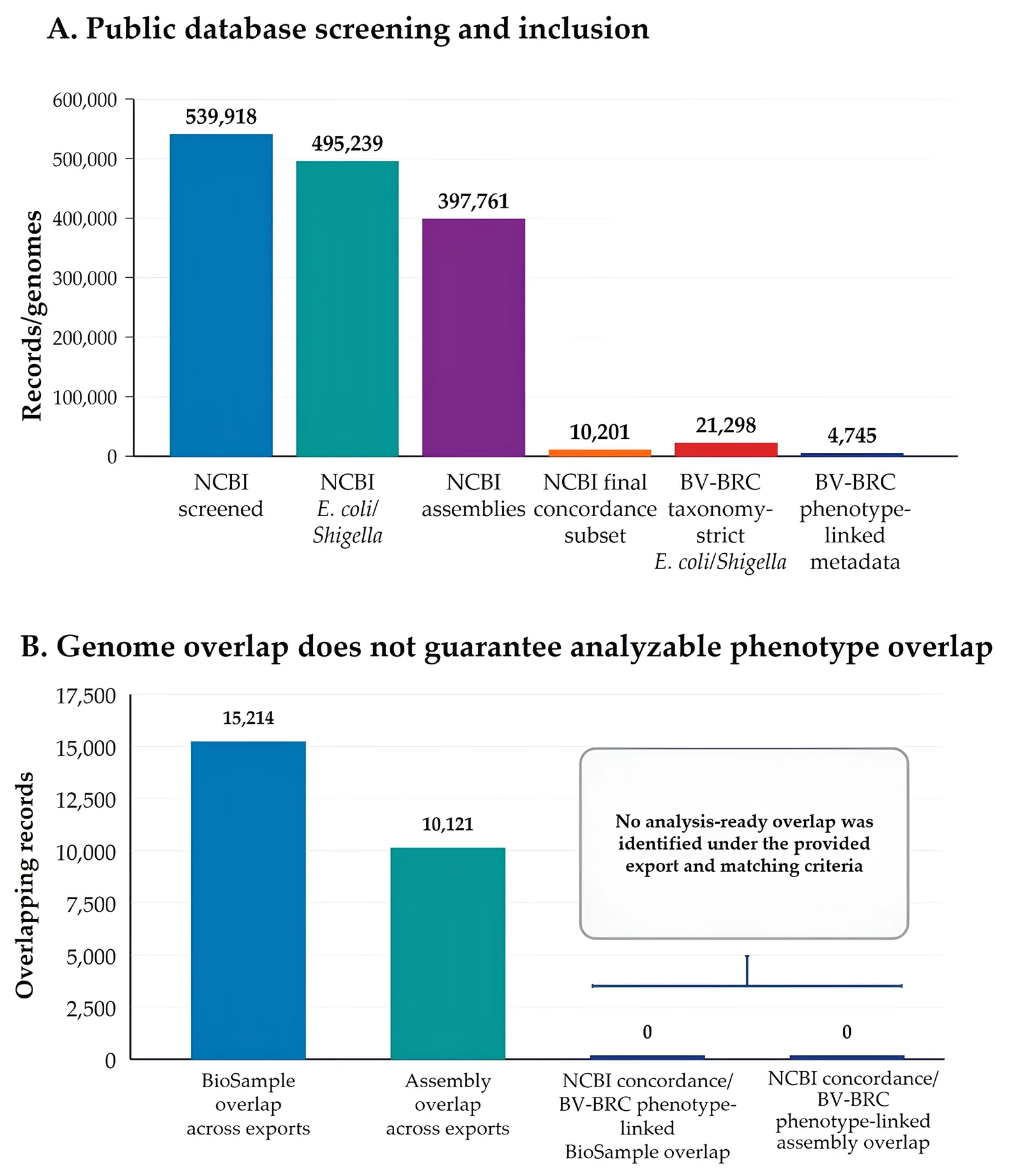
Public database screening and cross-database evidence linkage. The figure summarizes the analytical workflow and main findings from public *E. coli*/*Shigella* WGS-AMR metadata exported from NCBI Pathogen Detection and BV-BRC. Panel (**A**) shows database screening and inclusion, including NCBI records screened, retained *E. coli*/*Shigella* records, assembly-linked NCBI records, the final NCBI genotype–phenotype concordance subset, the taxonomy-strict BV-BRC *E. coli*/*Shigella* metadata cohort, and BV-BRC phenotype-linked metadata identifiers. Panel (**B**) distinguishes broad accession overlap from analysis-ready evidence overlap. Although 15,214 BioSample accessions and 10,121 assembly accessions were shared across the broad NCBI Pathogen Detection and BV-BRC exports, none remained shared between the final analysis-ready subsets after application of the database-specific genotype, phenotype, evidence-type, accession-linkage, and analytic eligibility criteria. The bottleneck, therefore, occurred in the availability and linkage of analytically compatible genotype–phenotype evidence rather than in genome-accession identity. Potential contributors include missing phenotype fields, differences in evidence type, non-overlapping antibiotic panels, incomplete identifier linkage, accession-version differences, and temporal differences between database exports. Accordingly, the absence of analysis-ready overlap should not be interpreted as evidence that corresponding isolates are absent from either database. This finding identifies a public-database interoperability gap and supports interpreting the resources as complementary rather than redundant.

**Figure 2:**
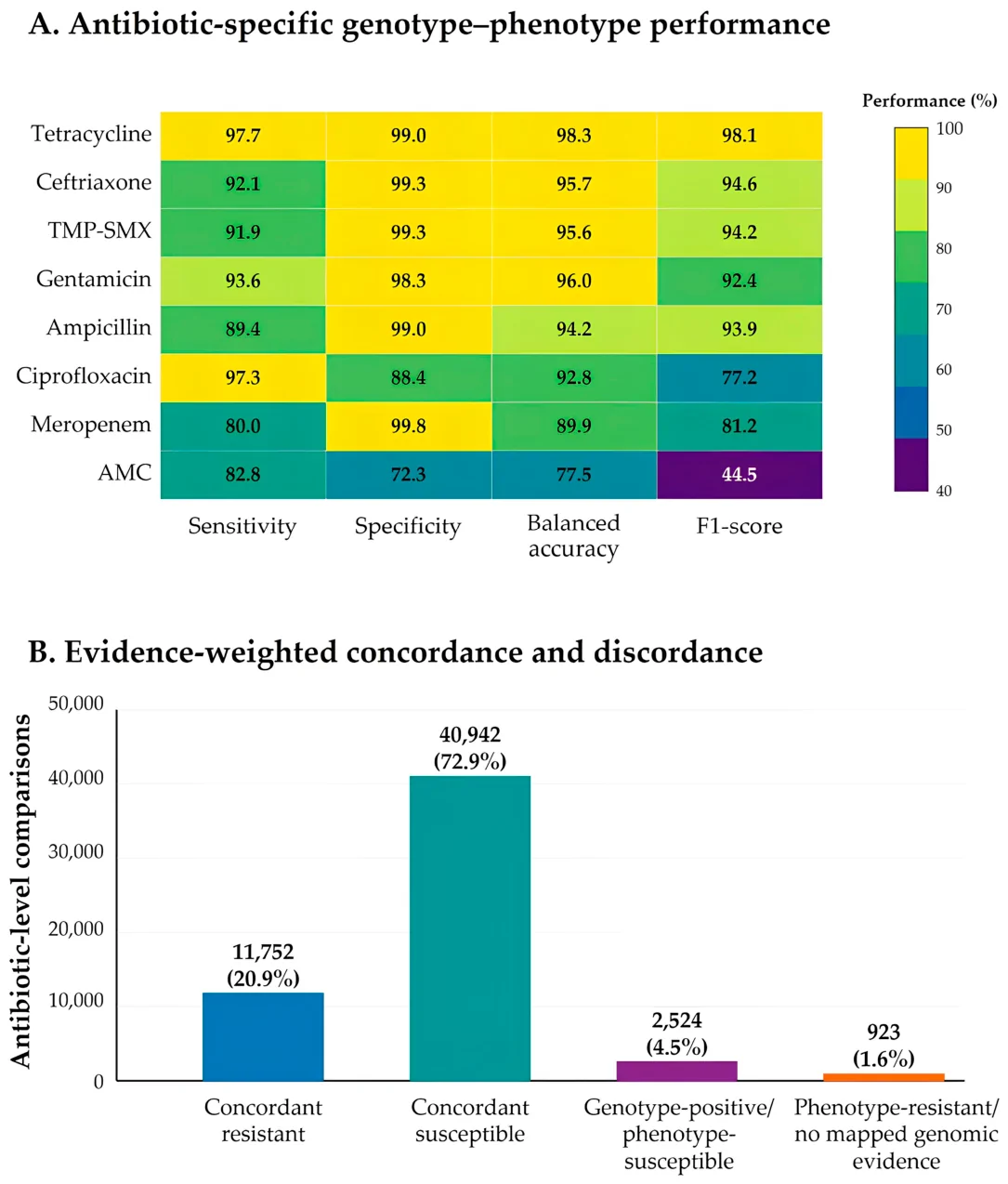
Antibiotic-specific genotype–phenotype performance and discordance across eight selected antibiotics. The figure summarizes antibiotic-specific performance and evidence-weighted discordance derived from the NCBI Pathogen Detection concordance subset. Panel (**A**) presents sensitivity, specificity, balanced accuracy, and F1-score for each of the eight priority antibiotics. Panel (**B**) shows evidence-weighted concordance and discordance categories across 56,141 resistant/susceptible genotype–phenotype comparisons, separating concordant resistant, concordant susceptible, genotype-positive/phenotype-susceptible discordance, and phenotype-resistant/no mapped genomic evidence discordance. Abbreviations: TMP-SMX, trimethoprim–sulfamethoxazole; AMC, amoxicillin–clavulanic acid.

A large-scale public database analysis was conducted using exported records from NCBI Pathogen Detection and BV-BRC. The database exports, metadata fields, evidence layers, and analytical roles used in the analyses are summarized in Supplementary Table S1. The NCBI Pathogen Detection export contained 539,918 raw records. After filtering for the public *E. coli*/*Shigella* organism group, 495,239 records were retained for analysis. These records were interpreted as a combined public database organism group, and the resulting estimates should not be interpreted as separate species-specific performance estimates for *E. coli or Shigella*. Among these, 397,761 records had assembly accessions, and 495,203 had exported AMR genotype annotations. AST phenotype information was available for 10,772 records, and 10,201 records contained the required combination of assembly accession, AMR genotype field, and interpretable AST phenotype data for genotype–phenotype concordance analysis. The BV-BRC genome export contained 22,854 total genome records. After application of the taxonomy-strict *E. coli*/*Shigella* rule, 21,298 genome records were retained, including 14,053 with assembly accessions. The BV-BRC AMR phenotype export contained 366,397 phenotype-related records linked to 4745 genome identifiers: all 4745 identifiers mapped to records classified as *E. coli* within the taxonomy-strict BV-BRC metadata cohort. Because the BV-BRC phenotype-related export included both laboratory and computational method records, it was used only as a complementary descriptive phenotype-related coverage and metadata-complementarity resource rather than as an external validation cohort. Laboratory method records were retained for laboratory-only summaries, whereas records labeled as Computational Method were treated as model-derived predictions and were not used as independent phenotypic evidence in the concordance analysis (Table 1 and Figure 1). Antibiotic-specific genotype–phenotype performance and evidence-weighted discordance across the eight selected antibiotics are summarized in Figure 2.

Geographic information was available for 10,138 of the 10,201 genotype–phenotype-eligible records. Among records with country data, the dataset was heavily weighted toward the United States (7298; 72.0%), followed by Canada (1334; 13.2%), the United Kingdom (581; 5.7%), the Netherlands (505; 5.0%), Mexico (154; 1.5%), and Germany (92; 0.9%); 63 records lacked a country assignment. Isolation-type metadata were available for 10,187 records, of which 6086 (59.7%) were classified as environmental/non-clinical and 4101 (40.3%) as clinical; 14 records lacked an isolation-type assignment. In the full analytical cohort, frequently reported isolation-source labels included urine (3347; 32.8%), ground turkey (933; 9.1%), a generic clinical category (581; 5.7%), ground beef (472; 4.6%), and blood (251; 2.5%). These distributions indicate substantial geographic and source imbalance and should be considered when evaluating generalizability.

### 2.2. Antibiotic Coverage and Interpretable AST Data in NCBI Pathogen Detection

Within the NCBI genotype–phenotype-eligible subset, AST entries were parsed and standardized across antibiotics. After excluding non-interpretable or missing values, 121,391 resistant/susceptible AST entries were available across 89 antibiotics. The antibiotics with the largest numbers of interpretable R/S observations were ciprofloxacin, ampicillin, gentamicin, tetracycline, trimethoprim–sulfamethoxazole, ceftriaxone, amoxicillin–clavulanic acid, and meropenem. These eight antibiotics were selected for the primary genotype–phenotype performance analysis because they represented clinically important antimicrobial classes and had sufficient sample sizes for stable antibiotic-specific comparison (Table 2).

### 2.3. Genotype–Phenotype Concordance Across Priority Antibiotics

Before genotype–phenotype performance was calculated, AMR genotype annotations were converted into antibiotic-specific classifications of genotype-positive resistance or no mapped genomic evidence of resistance using the predefined mapping rules. The resulting antibiotic-specific confusion matrices and extended performance metrics are provided in Supplementary Table S2, while the prespecified determinant-to-antibiotic mapping rules and supporting literature are summarized in Supplementary Table S3.

Across 56,141 antibiotic-level resistant/susceptible genotype–phenotype comparisons, 11,752 were concordant resistant, 40,942 were concordant susceptible, 2524 were genotype-positive/phenotype-susceptible discordances, and 923 were phenotype-resistant/no mapped genomic evidence discordances. Performance differed across antibiotics. Tetracycline showed the highest overall accuracy (98.4%) and F1-score (98.1%), whereas amoxicillin–clavulanic acid showed the lowest accuracy (73.6%) and F1-score (44.5%). Antibiotic-specific performance estimates are summarized in Table 3. Meropenem showed an overall accuracy of 99.6%; however, only 70 of the 6228 comparisons were phenotype-resistant, compared with 6158 phenotype-susceptible comparisons. The high accuracy was therefore strongly influenced by the predominance of susceptible observations. Meropenem sensitivity was 80.0%, specificity was 99.8%, balanced accuracy was 89.9%, and the F1-score was 81.2%. These metrics, together with the underlying TP, TN, FP, and FN counts, provide a more informative assessment of performance than accuracy alone for this class-imbalanced antibiotic dataset.

### 2.4. Discordance Analysis and Evidence-Weighted AMR Interpretation

Discordance analysis was used to identify cases where genotype-derived resistance classifications and AST phenotypes were inconsistent. Across the eight selected antibiotics, genotype-positive/phenotype-susceptible discordances were more common than phenotype-resistant observations with no mapped genomic evidence of resistance. This pattern indicates that a mapped resistance determinant was not invariably associated with a submitted resistant phenotype. Possible explanations include incomplete or poorly expressed genes, uncertain allele effects, breakpoint differences, heterogeneous AST methods, or metadata errors. Conversely, phenotype-resistant/no mapped genomic evidence discordance may reflect resistance mechanisms not captured in the genotype field, regulatory mechanisms, porin loss, efflux activation, chromosomal mutations not represented in the parsed rules, or variation in AST methodology. The evidence-weighted interpretation framework, therefore, classified each antibiotic-level comparison into four categories: concordant resistant, concordant susceptible/no mapped genomic evidence of resistance, genotype-positive but phenotype-susceptible discordance, and phenotype-resistant/no mapped genomic evidence discordance. This structure improves interpretability by separating high-confidence genotype–phenotype agreement from discordant cases that require additional biological or laboratory review (Table 4).

Amoxicillin–clavulanic acid accounted for 1518 of the 2524 genotype-positive/phenotype-susceptible discordances across the eight selected antibiotics (60.1%). Of the 2181 amoxicillin–clavulanic acid comparisons classified as genotype-positive, 1518 (69.6%) were phenotype-susceptible, and 663 (30.4%) were phenotype-resistant. Among the 1518 genotype-positive/phenotype-susceptible comparisons, TEM-family markers were present in 1081 (71.2%), with *bla*_TEM-1_ being predominant; it was identified in 1065 comparisons (70.2%) and was the sole matched marker in 949 comparisons (62.5%). CTX-M-family determinants were present in 462 discordant comparisons (30.4%), most frequently *bla*_CTX-M-15_ (184; 12.1%), *bla*_CTX-M-27_ (140; 9.2%), and *bla*_CTX-M-1_ (73; 4.8%). SHV-family markers occurred in 44 comparisons (2.9%), *bla*_OXA-1_ in 20 (1.3%), and AmpC-family markers in 12 (0.8%). In contrast, among the 663 genotype-positive/phenotype-resistant comparisons, AmpC-family markers were present in 529 (79.8%), primarily *bla*_CMY-2_ (490; 73.9%). No KPC-, NDM-, or OXA-48-like marker was identified among the genotype-positive/phenotype-susceptible comparisons. Because the determinant categories were non-mutually exclusive, individual comparisons could contribute to more than one family category. Detailed family- and marker-level findings are presented in Supplementary Table S4.

To assess the influence of the broad primary rule, a post hoc sensitivity analysis reclassified comparisons in which *bla*_TEM-1_ was the sole mapped amoxicillin–clavulanic acid determinant as having no mapped genomic evidence of resistance. The alternative rule was applied without reference to phenotype, and no isolate–antibiotic observations were removed. This reclassified 949 original false positives as true negatives and 37 original true positives as false negatives, yielding 626 true positives, 4913 true negatives, 569 false positives, and 175 false negatives while retaining all 6283 comparisons (801 resistant and 5482 susceptible phenotypes). Under the alternative rule, sensitivity was 78.2%, specificity was 89.6%, PPV was 52.4%, NPV was 96.6%, accuracy was 88.2%, balanced accuracy was 83.9%, and the F1-score was 62.7%. The F1-score improved but remained the lowest among the eight antibiotics; therefore, the post hoc analysis refined but did not overturn the primary conclusion (Supplementary Table S5).

### 2.5. Cross-Database Linkage and Descriptive BV-BRC Phenotype-Related Coverage

To evaluate whether public WGS-AMR resources provide comparable and reusable metadata, the NCBI Pathogen Detection dataset was compared with independently exported BV-BRC genome and AMR phenotype tables. The databases differed substantially in scale and metadata structure. NCBI Pathogen Detection contributed 495,239 *E. coli*/*Shigella* records and 397,761 assembly-linked records, whereas BV-BRC contributed 21,298 taxonomy-strict *E. coli*/*Shigella* genomes, including 14,053 with assembly accessions (Table 5).

The BV-BRC AMR phenotype-related export included 4745 phenotype-linked genome identifiers, all of which mapped to records labeled as *E. coli* within the taxonomy-strict metadata cohort. Records labeled as Laboratory Method were retained for optional laboratory-only summaries, whereas Computational Method records were treated as model-derived resistance predictions. Accordingly, BV-BRC was used for phenotype-related and metadata-complementarity assessment rather than as an independent AST validation dataset.

Although 15,214 BioSample accessions and 10,121 assembly accessions were shared between the provided NCBI Pathogen Detection and BV-BRC exports, these overlaps were identified by exact accession matching across the broad database exports before application of the analysis-specific evidence filters. The final NCBI concordance subset required the simultaneous availability of an assembly accession, an exported AMR genotype field, and an interpretable resistant or susceptible AST phenotype. The final BV-BRC subset required a phenotype-linked genome identifier within the taxonomy-strict metadata cohort, while computationally derived phenotype records were not treated as independent laboratory AST evidence. None of the shared assembly accessions simultaneously satisfied the final eligibility definitions applied to both frozen database snapshots. Thus, the bottleneck occurred at the level of linked and analytically compatible genotype–phenotype evidence rather than at the level of genome-accession identity. Potential contributors include missing phenotype fields, differences in evidence type, non-overlapping antibiotic panels, accession-version differences, incomplete identifier linkage, and temporal differences between database exports. Accordingly, the absence of analysis-ready overlap should not be interpreted as evidence that corresponding isolates are absent from either database.

Descriptive antibiotic-level phenotype-related coverage in BV-BRC is summarized in Table 6. The most frequently represented antibiotics included ceftazidime, ampicillin, ciprofloxacin, trimethoprim–sulfamethoxazole, meropenem, gentamicin, cefotaxime, piperacillin–tazobactam, cefuroxime, nalidixic acid, aztreonam, tobramycin, cefepime, cefazolin, and ceftriaxone. Several of these antibiotics overlapped with the eight-drug NCBI concordance analysis, allowing comparison of antibiotic representation and metadata availability across the two public resources. Because the BV-BRC export contained both Laboratory Method and Computational Method records and reflected non-random database submissions, the resistant-entry proportions shown in Table 6 were interpreted only as descriptive indicators of phenotype-related coverage and metadata structure. They should not be interpreted as laboratory resistance rates, population-prevalence estimates, or external validation results.

## 3. Discussion

This study shows that public WGS-AMR metadata can support large-scale genotype–phenotype concordance analysis when database source, accession linkage, phenotype availability, and antibiotic-specific inclusion criteria are handled transparently. Using NCBI Pathogen Detection, we identified a large *E. coli*/*Shigella* dataset with AMR genotype annotations and a smaller but analytically valuable subset with interpretable AST phenotype summaries. Across 56,141 antibiotic-level resistant/susceptible comparisons, genotype-field-based resistance inference showed strong concordance for several clinically important antibiotics, while also revealing antibiotic-specific discordance patterns that are critical for interpretation [3,8,36,37,38].

### 3.1. Antibiotic-Specific Genotype–Phenotype Performance and Biological Interpretation

Most antimicrobial classes were represented by only one antibiotic; therefore, the reported estimates should not be generalized to all agents within the corresponding class. Tetracycline represented the tetracycline class, gentamicin the aminoglycosides, trimethoprim–sulfamethoxazole the folate-pathway inhibitors, ceftriaxone the cephalosporins, amoxicillin–clavulanic acid the β-lactam/β-lactamase inhibitor combinations, and meropenem the carbapenems. The strongest performance was observed for individual antibiotics for which resistance is often captured by well-characterized acquired determinants or established resistance-associated mutations. Tetracycline showed the highest overall performance, while ceftriaxone, trimethoprim–sulfamethoxazole, gentamicin, and ampicillin also showed high concordance. In contrast, amoxicillin–clavulanic acid showed lower specificity and F1-score. This finding should be interpreted at the level of the individual antibiotic rather than as evidence of uniformly lower performance for all β-lactam/β-lactamase inhibitor combinations. More broadly, genotype-derived inference may be less reliable when the observed phenotype depends not only on the presence of a resistance determinant but also on enzyme expression, inhibitor susceptibility, promoter variation, porin alteration, efflux activity, regulatory changes, or interacting resistance mechanisms.

The combined *E. coli*/*Shigella* analysis was necessary because the public exports used in this study organized closely related records within a shared organism group and did not provide uniformly complete species-, pathotype-, or lineage-level assignments for every eligible record. The close genomic relationship between *E. coli* and *Shigella*, together with the difficulty of resolving some lineages using WGS-based taxonomic approaches, has been well documented [18,19]. Applying inconsistent submitted species labels retrospectively could therefore introduce additional taxonomic misclassification. Nevertheless, this aggregation may affect the reported estimates if the relative frequencies of resistance determinants, clonal lineages, exposure histories, ecological niches, or tested antibiotic panels differ between *Shigella* and the diverse commensal, intestinal pathogenic, and extraintestinal *E. coli* populations represented in the database. The resulting performance estimates should therefore be interpreted as weighted averages across the database-defined organism group and may conceal species-, pathotype-, or lineage-specific differences.

The present analysis was restricted to the public *E. coli*/*Shigella* organism group. However, previous studies indicate that genotype–phenotype concordance is influenced by the organism, antibiotic, resistance mechanism, and phenotype-definition framework. High concordance has been reported for several well-characterized resistance determinants in nontyphoidal *Salmonella*, including large isolate studies comparing WGS-derived resistance profiles with phenotypic AST [26,28]. Nevertheless, performance remained antibiotic dependent, and discordance persisted for resistance mechanisms that were incompletely represented or difficult to interpret genomically [28]. Similar organism- and drug-specific variation has been reported in *Campylobacter jejuni* and *Campylobacter coli*, where predictive performance differed according to the antimicrobial evaluated and the relative contribution of target-site mutations and acquired resistance genes [24]. Studies involving *Staphylococcus aureus* and vancomycin-resistant enterococci have likewise demonstrated that WGS can predict several resistance phenotypes reliably, although performance depends on the organism, the prediction tool, and the completeness of the underlying resistance catalog [39,40]. In *Klebsiella pneumoniae*, genomic prediction is additionally complicated by interactions among acquired β-lactamases, carbapenemases, porin alterations, efflux systems, and regulatory mechanisms [41]. More broadly, evaluations across multiple Gram-negative and Gram-positive species have shown that genotype–phenotype agreement varies among organism–antibiotic combinations and may be influenced by phenotype-interpretation criteria and sequence quality [42]. Collectively, these findings support the conclusion that concordance is not a universal property of WGS but an organism–antibiotic–mechanism-specific outcome.

The lower performance observed for amoxicillin–clavulanic acid is consistent with previous *E. coli* WGS studies reporting lower genotype–phenotype agreement for this agent than for several other antibiotics [14]. A recent multi-feature analysis of *E. coli* WGS data similarly identified amoxicillin–clavulanic acid as the lowest-performing antibiotic among the evaluated prediction tasks [43]. Discordance may reflect minimum inhibitory concentration (MIC) values close to interpretive breakpoints [14], method-dependent differences in phenotypic susceptibility testing and breakpoint application [44], and biological heterogeneity not captured by resistance-gene presence alone. Recent mechanistic evidence demonstrated that *bla*_TEM-1_ promoter variation, gene copy number, phylogenetic background, and lineage-dependent expression can influence co-amoxiclav MICs and, in some isolates, shift the phenotype across the clinical breakpoint [45]. Different resistance-gene databases and annotation pipelines may also produce different classifications because they differ in reference catalogs, sequence-identity and coverage thresholds, mutation content, gene-family hierarchies, and genotype-to-phenotype interpretation rules [4,33]. These findings support future evaluation of allele-aware, promoter, copy-number, expression-related, and lineage-context features. However, improved prediction should not be assumed without independent reanalysis against standardized reference AST and external validation in representative populations [11]. The present study evaluates resistance classifications derived from exported NCBI metadata and should not be interpreted as a comparative benchmark of alternative AMR databases or annotation algorithms.

Observed antibiotic-specific differences are biologically plausible, but this metadata-based analysis cannot assign individual discordant observations to a specific resistance mechanism because it relies on exported NCBI genotype annotations rather than independent reanalysis of raw sequence data. For ciprofloxacin, genotype–phenotype interpretation may be affected by the combination and clinical relevance of quinolone target mutations and plasmid-mediated quinolone-resistance determinants. For amoxicillin–clavulanic acid, the determinant-level analysis showed that genotype-positive/phenotype-susceptible discordance was driven predominantly by TEM-family and CTX-M-family annotations rather than by AmpC-family determinants. In particular, *bla*_TEM-1_ was identified in 70.2% of discordant comparisons and was the sole matched marker in 62.5%. The presence of a TEM- or SHV-family β-lactamase does not uniformly predict resistance to an inhibitor combination because the resulting phenotype may depend on exact allele identity, promoter strength, gene dosage, expression level, inhibitor susceptibility, and co-occurring permeability mechanisms. CTX-M-family genes were also common among discordant records, demonstrating that ESBL detection alone did not consistently predict the submitted amoxicillin–clavulanic acid phenotype. Conversely, AmpC-family annotations, particularly *bla*_CMY-2_, were concentrated among genotype-positive/phenotype-resistant comparisons. Nevertheless, detection of an AmpC-family gene does not establish AmpC overproduction because promoter, copy-number, and expression information were not independently assessed. This determinant distribution is consistent with the lower specificity, positive predictive value, and F1-score observed for amoxicillin–clavulanic acid and supports treating this antibiotic as a lower-confidence category for genotype-field-based inference [12,14,27]. Similarly, meropenem performance should be interpreted in the context of the low frequency of carbapenem-resistant phenotypes and the possibility that some non-carbapenemase mechanisms may not be fully represented in exported genotype fields.

The post hoc sensitivity analysis confirmed that the breadth of the prespecified mapping rule materially influenced amoxicillin–clavulanic acid performance. Reclassifying *bla*TEM-1-only comparisons increased specificity, PPV, accuracy, balanced accuracy, and F1-score, while sensitivity decreased. This finding does not establish that *bla*_TEM-1_ is irrelevant to the phenotype. Its effect may depend on promoter strength, copy number, expression, phylogenetic or lineage background, inhibitor susceptibility, and interacting permeability or regulatory mechanisms. The results instead show that simple gene-presence rules are insufficient for reliable inference of this phenotype and that the alternative rule remains exploratory rather than clinically validated.

### 3.2. Evidence-Weighted Interpretation of Genotype–Phenotype Discordance

The observed predominance of genotype-positive/phenotype-susceptible discordances over phenotype-resistant observations with no mapped genomic evidence of resistance provides an important finding beyond overall accuracy estimates [46]. This pattern may reflect incomplete genes, uncertain allele effects, gene silencing, low expression, breakpoint differences, phenotype metadata heterogeneity, or genotype rules that overpredict resistance for some antibiotic–determinant combinations. Reports of resistance-gene-positive but phenotypically susceptible *E. coli* further indicate that gene presence alone may not define the expressed antimicrobial phenotype [47]. Conversely, phenotype-resistant/no mapped genomic evidence discordances may indicate resistance mechanisms not represented in the genotype field, regulatory changes, porin loss, efflux activation, chromosomal mutations, or limitations in the exported metadata. Reporting both discordance directions separately is therefore essential for transparent AMR interpretation [32].

### 3.3. Complementarity and Interoperability of Public WGS-AMR Resources

The comparison between NCBI Pathogen Detection and BV-BRC demonstrates that major public WGS-AMR repositories should be regarded as complementary evidence systems rather than interchangeable or directly mergeable datasets. NCBI Pathogen Detection supported the primary genotype–phenotype concordance analysis because exported AMR genotype annotations and submitted AST phenotype summaries were available within the same metadata environment. In contrast, BV-BRC contributed additional information on phenotype-related antibiotic coverage, evidence-method classification, genome metadata structure, and accession organization [20,23,48]. This complementary role is important because public AMR resources differ substantially in their data models, curation procedures, evidence hierarchies, phenotype definitions, and reporting conventions. Consequently, the analytical value of a repository depends not only on the number of available genomes but also on whether genomic, phenotypic, methodological, and accession-level information can be linked in a form suitable for the intended analysis.

A particularly important contribution of BV-BRC was its explicit differentiation between records derived from Laboratory Method and Computational Method evidence. This distinction reinforces the principle that experimentally determined susceptibility phenotypes and model-derived resistance predictions represent different evidentiary layers and should not be treated as equivalent forms of validation. Computational predictions may be useful for annotation, prioritization, or hypothesis generation, but they do not provide independent phenotypic confirmation of resistance. Public AMR analyses must therefore preserve evidence provenance when combining or comparing records across databases. Failure to distinguish laboratory-derived and computationally inferred outcomes may inflate apparent agreement, introduce circularity into validation analyses, and obscure uncertainty in genotype-to-phenotype interpretation. More broadly, large public AMR-genomics resources can support surveillance-oriented and computational investigations only when database-specific evidence structures and analytical limitations are explicitly documented [49].

Although BioSample and assembly accession overlap was identified between the provided NCBI Pathogen Detection and BV-BRC exports, no analysis-ready overlap was identified between the NCBI primary genotype–AST concordance subset and the BV-BRC phenotype-linked subset after prespecified matching and eligibility filtering. This finding should be interpreted cautiously because it may reflect differences in export timing, database structure, phenotype availability, identifier linkage, metadata completeness, or evidence-layer distribution rather than the definitive absence of corresponding isolates across the two resources. Comparable challenges arising from incomplete and non-standardized metadata have been documented in public pathogen-genomics surveillance and can limit interpretation, integration, and secondary data reuse [34].

These findings identify an interoperability limitation at the level of analytically compatible evidence rather than at the level of genome availability. Similar challenges arising from incomplete, inconsistently structured, or non-standardized pathogen-genomics metadata have been recognized as major barriers to data integration, interpretation, and secondary reuse [34,35,49,50]. Public repositories are frequently developed for different operational purposes and may apply distinct approaches to organism classification, phenotype representation, antibiotic nomenclature, evidence labeling, duplicate handling, and missing-data encoding. Consequently, apparently comparable fields may differ semantically even when they share similar names. Successful cross-database integration therefore requires more than exact accession matching; it requires harmonization of database versions, export dates, taxonomic filters, phenotype categories, antibiotic names, evidence layers, accession identifiers, eligibility definitions, and missing-data rules [10,22,23,35].

The absence of analysis-ready overlap should therefore not be interpreted as evidence that corresponding isolates are absent from either database. Rather, it shows that the genotype and phenotype information required for a specific concordance analysis may be distributed unevenly across resources or represented through incompatible evidence structures. This distinction is critical because a negative linkage result may otherwise be misinterpreted as biological or epidemiological non-overlap when it is primarily a consequence of metadata architecture. Future public AMR infrastructures should prioritize persistent cross-repository identifiers, explicit evidence provenance, standardized antimicrobial nomenclature, harmonized phenotype fields, versioned database snapshots, and machine-readable links among genome assemblies, BioSamples, AST records, and resistance annotations. These improvements would increase the reproducibility of secondary analyses and enable more reliable integration of genomic and phenotypic surveillance data across platforms.

The comparison underscores the importance of cautiously interpreting resistance proportions obtained from public repositories. Neither NCBI Pathogen Detection nor BV-BRC represent a population-based sampling framework. Public databases may disproportionately include isolates submitted during outbreaks, targeted AMR investigations, food-safety surveillance, healthcare-associated transmission events, national sequencing initiatives, or studies focused on unusual or multidrug-resistant phenotypes. Geographic, clinical, environmental, and host-source representation may therefore be highly uneven. As a result, antibiotic-level resistant-entry proportions in BV-BRC should be interpreted as descriptive measures of submitted phenotype-related coverage rather than as estimates of resistance prevalence in a defined population [3,10,22,23,50]. The same caution applies to predictive values derived from the NCBI analytical subset because these measures depend on the resistance frequency and sampling composition of the selected public metadata cohort.

This study also supports the use of evidence-weighted interpretation for public AMR metadata. Simple binary reporting of genotype-positive or genotype-negative results may obscure biologically important uncertainty. By separating concordant resistant, concordant susceptible, genotype-positive/phenotype-susceptible, and phenotype-resistant/no mapped genomic evidence categories, the analysis provides a more transparent framework for interpreting WGS-AMR data. Such reporting is especially useful for surveillance, where discordant records may identify antibiotics, mechanisms, or metadata fields requiring further curation [12,25,27,33].

The findings have practical implications for future computational AMR studies. First, large public datasets can support informative surveillance analyses, but only after careful filtering, harmonization, and transparent assessment of sampling and metadata limitations [8]. Second, genotype–phenotype performance should be reported at the antibiotic level, not only as an overall metric, because performance differs substantially among antibiotics and organism–antibiotic combinations [28]. Third, cross-database comparisons should distinguish between accession overlap, phenotype availability, and actual analyzable genotype–phenotype overlap. Finally, submitted resistant-entry proportions from descriptive phenotype-related coverage tables should not be interpreted as population prevalence estimates because database submissions are non-random and are influenced by source composition, surveillance priorities, metadata completeness, and testing practices.

Taken together, the cross-database analysis shows that NCBI Pathogen Detection and BV-BRC provide distinct but complementary forms of value for genomic AMR research. NCBI Pathogen Detection offers a suitable structure for large-scale analysis of genotype annotations linked to submitted AST phenotypes, whereas BV-BRC provides broader contextual information on phenotype-related records, evidence methods, antibiotic representation, and metadata organization. BV-BRC should therefore be interpreted as a complementary metadata and phenotype-coverage resource rather than as an external validation cohort. More generally, public WGS-AMR databases should be integrated through an evidence-aware and database-aware framework that distinguishes accession overlap from analytical compatibility. Such an approach can prevent inappropriate pooling of heterogeneous records, improve transparency in secondary data analysis, and support more reliable reuse of public genomic resources for AMR surveillance.

### 3.4. Surveillance Implications and Future Directions

Future work should move from retrospective public metadata analysis toward prospectively validated, quality-assured WGS-AMR surveillance workflows [51]. Priority areas include independent reanalysis of raw assemblies using standardized AMR pipelines, routine reporting of software and database versions, linkage of genomic outputs with harmonized AST methods and interpretive breakpoints, source- and geography-stratified analyses, and One Health integration across human, animal, food, and environmental sectors [3,38,52,53,54]. Public WGS-AMR databases should also adopt clearer reporting standards for export dates, accession linkage, phenotype definitions, antibiotic panels, missing data rules, and genotype-to-phenotype interpretation assumptions [22,23]. These steps would improve reproducibility, reduce database-related bias, and support the translation of large-scale genomic AMR metadata into actionable surveillance evidence. Future studies should perform species-, pathotype-, and lineage-stratified analyses when sufficiently complete and quality-controlled taxonomic metadata are available. Such analyses could combine exact species assignments with sequence type, serotype, phylogenetic lineage, clinical or environmental source, geography, and time period. Separate performance estimates for *Shigella* lineages and major *E. coli* pathotypes would help determine whether the organism-group estimates remain stable across biologically and epidemiologically distinct subpopulations. Future comparative studies should apply the same transparent analytical framework to additional priority pathogens, including *Salmonella enterica*, *Klebsiella pneumoniae*, *Campylobacter* spp., *Pseudomonas aeruginosa*, *Staphylococcus aureus*, and *Enterococcus* spp. Such analyses should use organism-specific determinant catalogs, phenotype definitions, breakpoints, and genotype-to-antibiotic mapping rules rather than applying a single universal prediction framework across species.

Although tetracycline, ceftriaxone, trimethoprim–sulfamethoxazole, gentamicin, and ampicillin show strong concordance in the analyzed public metadata, these findings do not establish that phenotypic AST can be reduced or omitted for any antibiotic in clinical practice. This study was retrospective, database-derived, and not designed as a prospective clinical-validation or diagnostic-equivalence study. Clinical implementation would require prospective external validation against standardized MIC-based AST, current breakpoint-specific interpretation, predefined acceptable major- and very-major-error limits, independent validation populations, quality-control procedures, and assessment of turnaround time and clinical consequences.

In surveillance analyses, higher-concordance antibiotic–genotype combinations may provide more reliable aggregate summaries than lower-concordance combinations. However, the present study did not evaluate clinical workflows, treatment decisions, or prospective isolate prioritization. These genotype-derived classifications should not serve as the exclusive criterion for individual treatment selection. Discordant records should prompt review of exact allele identity, sequence quality, AST method and breakpoint, gene completeness, promoter or expression effects, porin and efflux mechanisms, and possible metadata errors. Lower-confidence categories—particularly amoxicillin–clavulanic acid—and rare resistant phenotypes, such as meropenem resistance in this dataset, require continued phenotypic confirmation.

Overall, the findings support interpreting public WGS-AMR resources as complementary evidence systems. NCBI Pathogen Detection is particularly valuable for genotype-linked concordance analysis, whereas BV-BRC provides complementary descriptive phenotype-related coverage and metadata context. Together, these resources can support surveillance-oriented computational AMR research when their limitations are explicitly acknowledged and their outputs are interpreted using transparent, evidence-weighted rules [11,52].

### 3.5. Limitations

This study has several limitations that should be considered when interpreting the findings. First, the analysis relied on public database exports, and the results depend on the completeness, accuracy, and consistency of submitted metadata, including AST methods, interpretive breakpoints, isolate sources, geographic information, sampling strategies, and database update status. In addition, PPV and NPV were calculated within a non-random public-database cohort and were dependent on the resistance frequency represented in that cohort. These estimates should therefore not be transferred directly to clinical or population settings with different sampling structures or resistance distributions. Second, genotype–phenotype concordance was based on AMR genotype annotations exported from NCBI Pathogen Detection; therefore, this study should be interpreted as a real-world public WGS-AMR metadata concordance analysis rather than as an independent benchmark of AMR detection tools. Third, only interpretable resistant or susceptible AST phenotype entries were included in the primary binary concordance analysis, whereas intermediate, missing, non-standard, or non-interpretable phenotype entries were excluded, which may have influenced the final analytic subset. Fourth, genotype-derived resistance classifications were based on predefined antibiotic-specific mapping rules, and some mechanisms, including regulatory variation, porin loss, efflux activation, promoter effects, copy-number variation, gene-expression differences, mobile-element context, or novel resistance mechanisms, may not be captured in exported genotype fields [41]. For amoxicillin–clavulanic acid, the sensitivity analysis was post hoc and evaluated only one alternative rule in which *bla*_TEM-1_ alone was not sufficient for genotype-positive classification. It does not establish an optimal or clinically validated mapping framework and should not be interpreted as evidence that *bla*_TEM-1_ is phenotypically irrelevant. Allele-specific function, promoter variation, expression, copy number, porin status, and other genomic context could not be comprehensively evaluated in the exported metadata. Fifth, BV-BRC was included as a complementary metadata resource rather than as an external validation cohort. Its phenotype-related records contained heterogeneous evidence layers, including Laboratory Method and Computational Method entries. Laboratory-only summaries were generated where relevant, whereas computational predictions and functional annotations were excluded from the primary concordance and classification-performance analyses. Sixth, the absence of analysis-ready overlap after prespecified matching and eligibility filtering between NCBI Pathogen Detection and BV-BRC applies to the provided exports and may change with different export dates, database updates, or alternative filtering strategies [55]. The two resources were also exported on different dates: 11 June 2026 for NCBI Pathogen Detection and 14 June 2026 for BV-BRC. Although this three-day interval was short, continuously updated records, accession links, phenotype entries, or annotation rules could theoretically change between snapshots. A fixed-date design improves reproducibility because the input records and analytical denominators can be frozen and archived; however, it provides a historical snapshot rather than a continuous current representation of either database. A rolling analysis would improve currency but would require scheduled versioned exports, automated data-quality checks, and explicit reporting of changes in record counts, annotation pipelines, phenotype fields, and accession linkage at every update. The reported findings should therefore be interpreted as applying to the frozen June 2026 database snapshots and may not reproduce exactly from current or future live database contents. Seventh, the analysis was conducted at the combined *E. coli*/*Shigella* organism-group level used in public database exports and should not be interpreted as providing species-, pathotype-, or lineage-specific estimates. Eighth, the analytical cohort was geographically and compositionally imbalanced, with strong representation from the United States and selected clinical, food-animal, and food-product sources. Consequently, the antibiotic-specific estimates may partly reflect the lineages, testing practices, surveillance programs, and resistance distributions represented by these submissions. Finally, this surveillance-oriented computational study does not replace phenotypic AST, expert microbiological review, prospective clinical validation, or independent reanalysis of assemblies using standardized AMR pipelines [3,51,56]. Future studies should validate these estimates using independently processed assemblies, harmonized AST methods and breakpoints, and prospectively curated datasets. Where sample sizes permit, performance should be stratified by species, lineage, geography, isolation source, and time.

## 4. Materials and Methods

### 4.1. Study Design

This study was designed as a retrospective computational genomic epidemiology analysis of publicly available WGS-AMR metadata. The primary analysis used NCBI Pathogen Detection records to evaluate genotype–phenotype concordance in the *E. coli*/*Shigella* organism group. BV-BRC was used as a complementary metadata and descriptive phenotype-related coverage resource to evaluate genome metadata structure, antibiotic representation, accession overlap, and AMR phenotype-related coverage. The primary analysis was intentionally restricted to the public *E. coli*/*Shigella* organism group. Other bacterial pathogens were not included because genotype-to-phenotype inference is organism–antibiotic-specific and would require separate taxonomic filtering, phenotype harmonization, breakpoint interpretation, resistance-determinant mapping rules, and performance evaluation. Accordingly, this study was not designed as a cross-species comparison of genotype–phenotype concordance.

### 4.2. Data Collection, Database Snapshots, and Data Sources

In this study, two frozen public-database exports were analyzed. NCBI Pathogen Detection data were exported on 11 June 2026, and the BV-BRC genome and AMR phenotype-related tables were exported on 14 June 2026. Within the final NCBI genotype–phenotype-eligible subset, the exported metadata reported AMRFinderPlus version 4.2.7 for all 10,201 records. These dates define the database snapshots used throughout the analysis. The original exported files were retained without subsequent updating, and all derived analyses were generated from these frozen snapshots. The NCBI Pathogen Detection export provided organism metadata, assembly-accession information, exported AMR genotype annotations, and AST phenotype summaries. It was used for the primary genotype–phenotype concordance analysis because genotype and phenotype fields were available within the same metadata source. The BV-BRC genome and AMR phenotype-related exports were used for complementary cross-database comparison of genome counts, assembly accession availability, descriptive AMR phenotype-related coverage, antibiotic representation, and overlap with NCBI records by BioSample and assembly accession [20,21,48]. The public exports did not provide complete identifiers for every internal software build, allele catalog, annotation database, or genotype-to-phenotype mapping version used by the respective platforms. Therefore, reproducibility was anchored to the exact export dates, frozen input files, database source, organism filters, and supplementary processing rules. Because public databases and their underlying annotation pipelines are updated over time, future exports may produce different record counts, allele assignments, or automated AMR classifications.

### 4.3. Inclusion and Exclusion Criteria

Records were included if they belonged to the *E. coli*/*Shigella* organism group in the exported public database metadata. This grouping reflects the close genomic relationship between *E. coli* and *Shigella* and the recognized difficulty of distinguishing some lineages using sequence-based classification alone [18,19]. The primary NCBI genotype–phenotype findings were therefore interpreted at the organism-group level rather than as species-, pathotype-, or lineage-specific estimates.

For BV-BRC, a taxonomy-strict metadata cohort was defined separately using records assigned to *E. coli* or to the genus *Shigella*. BV-BRC phenotype-linked identifiers were then matched back to this strict metadata cohort. The phenotype-linked BV-BRC subset used for descriptive antibiotic coverage mapped to exact *E. coli* records and was therefore interpreted as a descriptive *E. coli* phenotype-linked subset, not as a combined *E. coli*/*Shigella* phenotype dataset.

### 4.4. Data Cleaning and Harmonization

Database fields were cleaned and standardized before analysis. Organism names, assembly accessions, BioSample identifiers, antibiotic names, antibiotic classes, AMR genotype fields, and phenotype categories were harmonized. Antibiotic names were standardized to common labels to avoid duplicate representation caused by spelling, capitalization, or formatting differences. Phenotype results were categorized as resistant, susceptible, or intermediate when available. The primary genotype–phenotype concordance analysis used resistant and susceptible entries only.

### 4.5. NCBI AST Phenotype Parsing

NCBI AST phenotype summaries were converted into a long-format isolate–antibiotic table. Each row represented one isolate–antibiotic comparison with an interpretable resistant or susceptible phenotype. Antibiotics with the largest interpretable R/S sample sizes were identified. The eight antibiotics selected for the primary concordance analysis were ciprofloxacin, ampicillin, gentamicin, tetracycline, trimethoprim–sulfamethoxazole, ceftriaxone, amoxicillin–clavulanic acid, and meropenem. Antibiotics outside the primary eight-drug analysis were retained in the source-data package and supplementary coverage summaries but were not included in the main concordance analysis because their smaller or less consistent interpretable R/S sample sizes would have reduced the stability and comparability of antibiotic-specific performance estimates. NCBI AST phenotype entries were treated as submitted public metadata and may have differed from genotype-derived AMR calls. The present analysis did not independently validate the underlying AST methods, interpretive breakpoints, or reported values. Duplicate isolate–antibiotic records were identified using the isolate identifier and standardized antibiotic name. Exact duplicate rows were collapsed before analysis. Residual non-identical records sharing an isolate–antibiotic key were retained as submitted, and their within-isolate dependence was preserved through isolate-level bootstrap resampling. Resistant and susceptible classifications were analyzed as submitted because AST method, MIC or inhibition-zone value, interpretive standard, breakpoint version, and testing date were not consistently available. Consequently, phenotypes could not be retrospectively harmonized to a single CLSI or EUCAST standard.

### 4.6. NCBI AMR Genotype Parsing and Genotype-Derived Prediction Rules

AMR genotype annotations exported from NCBI Pathogen Detection were parsed and mapped to antibiotic-specific genotype-derived resistance classifications. The frozen NCBI export reported AMRFinderPlus version 4.2.7 for all 10,201 genotype–phenotype-eligible records. No independent reanalysis of raw reads or assemblies was undertaken, and no additional AMR detection software was applied. Genotype-derived resistance classifications were generated by matching resistance determinants to the antibiotic or antimicrobial class to which they were mapped under the prespecified antibiotic-specific rules in Supplementary Table S3. Because AMR genotype annotations were obtained from the NCBI Pathogen Detection metadata export, the analysis depends on the database annotation pipeline, database versioning, and update status at the time of export [21,33].

### 4.7. Antibiotic-Specific Genotype-to-Phenotype Mapping Rules

To generate genotype-derived resistance classifications, AMR determinants reported in the NCBI Pathogen Detection AMR genotype field were mapped to antibiotic-specific resistance categories using a predefined genotype-to-phenotype interpretation framework [33,42]. The mapping was performed at the antimicrobial-class and resistance-mechanism level rather than by relying solely on exact gene names, because AMRFinderPlus and related genotype-to-phenotype frameworks classify resistance determinants by acquired genes, resistance-associated point mutations, antimicrobial class, and predicted phenotype, while public AMR metadata may also include allele-level variation, gene-family labels, and database-specific nomenclature.

A genotype-positive classification was assigned when the exported genotype field contained at least one resistance determinant mapped to the tested antibiotic under the prespecified rules reported in Supplementary Table S3. If a mapped determinant was absent, the isolate–antibiotic comparison was classified as lacking mapped genomic evidence of resistance for the tested antibiotic. For brevity in statistical definitions, this category corresponds to a genotype-negative classification for the antibiotic-specific mapping rule; it does not indicate the absence of all resistance genes from the isolate. This classification indicates the absence of a mapped determinant in the exported NCBI AMR genotype field and should not be interpreted as definitive biological susceptibility.

For β-lactam antibiotics, β-lactamase determinants were mapped according to their expected spectrum, including narrow-spectrum penicillinases, extended-spectrum β-lactamases, AmpC-type β-lactamases, and carbapenemases. For aminoglycosides, aminoglycoside-modifying enzymes and 16S rRNA methyltransferases were considered, with interpretation restricted to determinants known or expected to affect the relevant aminoglycoside phenotype. For fluoroquinolones, plasmid-mediated quinolone resistance determinants and resistance-associated quinolone target mutations were considered when explicitly present in the exported genotype field. For tetracycline, *tet*-family determinants were mapped to tetracycline resistance. For trimethoprim–sulfamethoxazole, *dfr* and *sul* determinants were mapped according to the prespecified antibiotic-specific rule. All determinant inclusions, exclusions, and interpretation notes are reported in Supplementary Table S3.

The genotype-to-antibiotic mapping framework was defined before calculating concordance metrics and was applied uniformly across all eligible isolate–antibiotic comparisons [12,25]. No antibiotic-specific rule was modified after inspecting genotype–phenotype performance. The analysis did not infer resistance from raw sequence data and did not independently rerun external AMR detection tools. Therefore, genotype-derived resistance classifications should be interpreted as NCBI metadata-based resistance inferences rather than de novo genomic predictions from raw reads or assemblies. The full antibiotic-specific mapping framework is provided in Supplementary Table S3.

Because amoxicillin–clavulanic acid showed the lowest specificity, PPV, and F1-score among the eight selected antibiotics, a post hoc descriptive determinant-level analysis was performed for this lower-confidence category. Genotype-positive/phenotype-susceptible and genotype-positive/phenotype-resistant comparisons were identified in the final isolate–antibiotic prediction table. Exact-match β-lactamase determinants were extracted from the exported NCBI AMR genotype field and grouped into TEM-, SHV-, CTX-M-, AmpC-, OXA-1-, OXA-48-like carbapenemase-, other major carbapenemase-, other OXA-, and other β-lactamase categories. Determinant-family categories were treated as non-mutually exclusive because individual comparisons could contain more than one matched β-lactamase marker. Frequencies were reported separately for genotype-positive/phenotype-susceptible and genotype-positive/phenotype-resistant comparisons. This exploratory analysis did not modify the predefined mapping rules or the primary antibiotic-level performance estimates. AmpC-family gene detection was reported as a genomic annotation only; AmpC overproduction was not inferred because promoter activity, gene copy number, and expression data were unavailable in the exported metadata.

A post hoc sensitivity analysis then evaluated a more restrictive amoxicillin–clavulanic acid rule in which *bla*_TEM-1_ was not sufficient for genotype-positive classification when it was the sole mapped determinant. The alternative classification was applied independently of phenotype. For this analysis, *bla*TEM-1-only was operationally defined as a comparison in which *bla*_TEM-1_ was the only mapped acquired β-lactamase marker and the exported genotype field containing no TEM-family promoter mutation annotated as POINT. Comparisons containing *bla*_TEM-1_ together with another mapped determinant remained genotype-positive, no isolate–antibiotic observations were removed, and the resistant and susceptible phenotype denominators remained unchanged. Performance metrics were recalculated using the definitions above, and uncertainty was estimated using the same isolate-level percentile-bootstrap procedure with 10,000 replicates and fixed random seed 20260611. The primary prespecified analysis was retained unchanged and is reported separately from this exploratory alternative-rule analysis.

### 4.8. Genotype–Phenotype Concordance Metrics and Statistical Analysis

For each antibiotic, genotype-derived resistance classifications were compared with resistant/susceptible AST phenotypes using a two-by-two confusion matrix. True positives were defined as genotype-positive and phenotype-resistant comparisons; true negatives were comparisons with no mapped genomic evidence of resistance and phenotype-susceptible AST results; false positives were genotype-positive but phenotype-susceptible comparisons; and false negatives were phenotype-resistant comparisons with no mapped genomic evidence of resistance.

For each antibiotic, sensitivity, specificity, positive predictive value (PPV), negative predictive value (NPV), accuracy, balanced accuracy, and F1-score were calculated. Sensitivity was calculated as TP/(TP + FN), specificity as TN/(TN + FP), PPV as TP/(TP + FP), NPV as TN/(TN + FN), accuracy as (TP + TN)/(TP + TN + FP + FN), and balanced accuracy as the mean of sensitivity and specificity. The F1-score, defined as the harmonic mean of PPV and sensitivity, was calculated as 2 × TP/(2 × TP + FP + FN).

Uncertainty was estimated using a nonparametric isolate-level bootstrap with 10,000 replicates. For each antibiotic, isolate identifiers were sampled with replacement from the corresponding final analysis subset, and all isolate–antibiotic observations associated with each sampled identifier were retained. Performance metrics were recalculated for each bootstrap sample. Two-sided 95% confidence intervals were obtained using the percentile method, defined by the 2.5th and 97.5th percentiles of the bootstrap distribution. A fixed random seed of 20260611 was used to support computational reproducibility. Isolate-level rather than individual-row resampling was used to preserve any dependence among repeated entries belonging to the same isolate.

Because accuracy may be inflated when susceptible phenotypes dominate, particularly for antibiotics with low resistance frequency, such as meropenem, performance interpretation prioritized the full TP/TN/FP/FN distribution together with sensitivity, specificity, PPV, NPV, balanced accuracy, and F1-score rather than accuracy alone. Table 3 presents point estimates and bootstrap-derived 95% confidence intervals for all performance metrics displayed. Supplementary Table S2 provides the complete TP, TN, FP, and FN counts; resistant and susceptible AST counts; point estimates for all performance metrics; and confidence intervals for sensitivity, specificity, accuracy, and F1-score.

### 4.9. Cross-Database Comparison with BV-BRC

NCBI Pathogen Detection and BV-BRC were compared by export size, organism-group retention, assembly accession availability, NCBI AMR genotype and AST phenotype fields, BV-BRC AMR phenotype-related metadata, and BioSample and assembly accession overlap. The taxonomy-strict BV-BRC cohort included records whose standardized organism field was assigned to *E. coli* or to a species within the genus *Shigella*. Records containing ambiguous, environmental, mixed-organism, or higher-level taxonomic assignments were excluded. BV-BRC phenotype-linked identifiers were separately matched to this strict metadata cohort. BV-BRC AMR phenotype-related entries were harmonized by antibiotic, phenotype category, unique genome identifier, and evidence type. Laboratory Method records were retained as a laboratory-only coverage subset, whereas Computational Method records were classified as model-derived predictions. The combined phenotype-related dataset was used only for descriptive coverage and metadata-complementarity analyses. BV-BRC specialty-gene, subsystem, and pathway annotation files were retained as descriptive functional-context resources and were not included in the primary concordance or classification-performance calculations (Table 6).

### 4.10. Ethics Statement

This study used publicly available pathogen-genomics metadata and did not involve direct contact with human participants, identifiable private information, animal intervention, or new biological sampling. Therefore, institutional review board approval and informed consent were not required.

### 4.11. Reproducibility and Reporting

To support reproducibility, all analyses reported database source, export date, organism filters, accession-linkage rules, phenotype definitions, antibiotic harmonization rules, missing data criteria, and genotype-to-antibiotic mapping assumptions. The database export date was treated as an essential reproducibility variable because the underlying public records, resistance-gene catalogs, allele assignments, and automated annotation rules may change between database releases. Cleaned tables, processed analysis outputs, genotype-to-antibiotic mapping rules, database-specific source-data packages, and supplementary data dictionaries are provided in the Supplementary Materials.

Data cleaning, phenotype parsing, antibiotic-name harmonization, genotype-to-antibiotic mapping, concordance classification, statistical analysis, and figure generation were performed using custom Python scripts in Python version 3.13.5 with pandas version 2.2.3. Concordance analysis was implemented by assigning each isolate–antibiotic comparison to a two-by-two confusion-matrix category—true positive, true negative, false positive, or false negative—according to the prespecified antibiotic-specific mapping rules and the submitted resistant/susceptible AST phenotype. Sensitivity, specificity, PPV, NPV, accuracy, balanced accuracy, and F1-score were calculated directly from these confusion-matrix counts. Two-sided 95% confidence intervals were estimated using 10,000 nonparametric isolate-level bootstrap replicates with percentile intervals and a fixed random seed of 20260611. The post hoc sensitivity analysis was rerun in Python 3.12.13 with pandas 2.2.3 and NumPy 2.3.5; the random-number generator was NumPy default_rng (PCG64). No independent AMR detection software was rerun on raw reads or assemblies; the genotype input consisted of AMR annotations exported from NCBI Pathogen Detection. The analytical scripts, README file, environment and package specifications, and processed output tables are provided in the Supplementary Data, as described in Appendix A.1 and Appendix A.2.

## 5. Conclusions

NCBI Pathogen Detection metadata supported large-scale, antibiotic-specific evaluation of concordance between exported AMR genotype annotations and submitted AST phenotypes in the database-defined *E. coli*/*Shigella* organism group. Concordance was high for several antibiotics but substantially lower for amoxicillin–clavulanic acid, demonstrating that genotype-derived classification performance cannot be generalized across antimicrobial agents. Separating genotype-positive/phenotype-susceptible from phenotype-resistant/no mapped genomic evidence discordance provided greater interpretability than aggregate accuracy alone. The cross-database analysis further showed that broad accession overlap between NCBI Pathogen Detection and BV-BRC did not yield analysis-ready genotype–phenotype linkage after evidence-specific filtering. Public WGS-AMR resources can therefore support surveillance-oriented secondary analyses when database snapshots, evidence provenance, phenotype definitions, accession linkage, and antibiotic-specific mapping rules are reported transparently. These database-derived estimates do not constitute clinical diagnostic validation and should not replace standardized phenotypic AST. The post hoc analysis further showed that the breadth of the amoxicillin–clavulanic acid mapping rule materially affected performance; however, the alternative rule remained insufficient for clinical inference without phenotype-linked validation.

## Figures and Tables

**Table 1:** Screening and derivation of the analytical cohorts from NCBI Pathogen Detection and BV-BRC.

Screening/Inclusion Level	Data Source	Number of Records or Genomes	Role in the Analysis
Raw records screened	NCBI Pathogen Detection	539,918	Initial public AMR-genomics dataset
*E. coli*/*Shigella* records retained	NCBI Pathogen Detection	495,239	Main organism group for the primary analysis
Records with assembly accessions	NCBI Pathogen Detection	397,761	Eligible for assembly-linked AMR metadata analysis
Records with exported AMR genotype annotations	NCBI Pathogen Detection	495,203	Source field for genotype-derived AMR inference
Records with an AST phenotype field	NCBI Pathogen Detection	10,772	Candidate genotype–phenotype records
Records with assembly accession, AMR genotype field, and interpretable AST phenotype	NCBI Pathogen Detection	10,201	Final subset for genotype–phenotype concordance analysis
Total genome records in the provided export	BV-BRC	22,854	Initial BV-BRC genome metadata export
*E. coli*/*Shigella* genomes retained	BV-BRC	21,298	Taxonomy-strict BV-BRC metadata cohort
Genomes with assembly accessions	BV-BRC	14,053	Assembly-linked metadata comparison
Genomes with AMR phenotype-related metadata	BV-BRC	4745	Complementary phenotype-linked metadata subset; all phenotype-linked IDs matched exact *E. coli* records

**Note:** NCBI Pathogen Detection was used for the primary genotype–phenotype concordance analysis because genotype annotations and AST phenotype summaries were available within the same metadata source. BV-BRC was used to characterize phenotype-related coverage, evidence-type structure, and cross-database metadata complementarity; it was not treated as an external validation cohort.

**Table 2:** Eight antibiotics selected for the primary NCBI genotype–phenotype concordance analysis.

Antibiotic	Antibiotic Class	Interpretable R/S AST Comparisons
Ciprofloxacin	Fluoroquinolone	7615
Ampicillin	Penicillin/β-lactam	7592
Gentamicin	Aminoglycoside	7344
Tetracycline	Tetracycline	7203
Trimethoprim–sulfamethoxazole	Folate-pathway inhibitor	7035
Ceftriaxone	Cephalosporin/β-lactam	6841
Amoxicillin–clavulanic acid	β-lactam/β-lactamase inhibitor	6283
Meropenem	Carbapenem/β-lactam	6228

**Table 3:** Genotype–phenotype classification performance for the antibiotics selected from NCBI Pathogen Detection.

Antibiotic	N R/S AST	Sensitivity	Specificity	Accuracy	PPV	NPV	Balanced Accuracy	F1-Score
Ciprofloxacin	7615	97.3% (96.4–98.2)	88.4% (87.6–89.2)	89.9% (89.3–90.6)	63.9% (61.8–66.0)	99.4% (99.1–99.6)	92.8% (92.2–93.4)	77.2% (75.5–78.7)
Ampicillin	7592	89.4% (88.4–90.4)	99.0% (98.7–99.3)	94.5% (94.0–95.0)	98.7% (98.4–99.2)	91.5% (90.5–92.2)	94.2% (93.7–94.7)	93.9% (93.3–94.4)
Gentamicin	7344	93.6% (92.1–94.9)	98.3% (98.0–98.7)	97.6% (97.2–97.9)	91.1% (89.6–92.8)	98.8% (98.5–99.1)	96.0% (95.2–96.6)	92.4% (91.2–93.5)
Tetracycline	7203	97.7% (97.1–98.2)	99.0% (98.6–99.2)	98.4% (98.1–98.7)	98.6% (98.2–99.0)	98.3% (97.8–98.6)	98.3% (98.0–98.6)	98.1% (97.8–98.5)
Trimethoprim–sulfamethoxazole	7035	91.9% (90.4–93.4)	99.3% (99.0–99.5)	97.9% (97.6–98.3)	96.8% (95.5–97.5)	98.1% (97.9–98.6)	95.6% (94.9–96.4)	94.2% (93.2–95.1)
Ceftriaxone	6841	92.1% (90.7–93.5)	99.3% (99.1–99.5)	97.9% (97.5–98.2)	97.3% (96.3–98.1)	98.0% (97.6–98.4)	95.7% (95.0–96.4)	94.6% (93.7–95.4)
Amoxicillin–clavulanic acid	6283	82.8% (80.0–85.3)	72.3% (71.2–73.5)	73.6% (72.6–74.7)	30.4% (28.5–32.4)	96.6% (96.1–97.2)	77.5% (76.1–78.9)	44.5% (42.2–46.6)
Meropenem	6228	80.0% (70.3–88.9)	99.8% (99.7–99.9)	99.6% (99.4–99.7)	82.4% (73.0–91.3)	99.8% (99.6–99.9)	89.9% (85.0–94.4)	81.2% (73.0–87.5)

**Table note:** Values are percentages with two-sided 95% percentile-bootstrap confidence intervals in parentheses, calculated from 10,000 isolate-level nonparametric bootstrap replicates. Full TP, TN, FP, and FN counts; resistant and susceptible AST counts; and point estimates for all performance metrics are provided in Supplementary Table S2. Confidence intervals for sensitivity, specificity, accuracy, and F1-score are also reported in Supplementary Table S2. Table 3 reports the prespecified primary analysis; the post hoc alternative amoxicillin–clavulanic acid analysis is reported separately in Section 2.4 and Supplementary Table S5.

**Table 4:** Evidence-weighted genotype–phenotype outcome categories across the eight priority antibiotics.

Evidence Category	Count	Interpretation
Concordant resistant/true positive	11,752	Classified genotype-positive for resistance, and AST was resistant
Concordant susceptible/true negative	40,942	No mapped genomic evidence of resistance, and AST was susceptible
Genotype-positive/phenotype-susceptible discordance (statistical false positive)	2524	Resistance determinant detected, but AST was susceptible
Phenotype-resistant/no mapped genomic evidence discordance (statistical false negative)	923	AST was resistant, but no antibiotic-specific resistance determinant mapped by the prespecified rule was identified in the exported genotype field
Total interpretable genotype–phenotype comparisons	56,141	All R/S comparisons across the eight selected antibiotics

**Table 5:** Cross-database comparison of NCBI Pathogen Detection and BV-BRC AMR datasets.

Metric	NCBI Pathogen Detection	BV-BRC	Cross-Database Result
Raw records/genomes in the provided export	539,918	22,854	-
*E. coli*/*Shigella* target cohort retained	495,239	21,298	-
Records/genomes with assembly accession	397,761	14,053	-
Records with exported AMR genotype annotations	495,203	Not applicable	-
Records/genomes with phenotype-related metadata	10,772 records with an AST phenotype field	4745 phenotype-linked genome identifiers, all exact *E. coli*	-
Final primary concordance subset/descriptive phenotype-related metadata subset	10,201	4745	-
Shared BioSample accessions across exports	-	-	15,214
Shared assembly accessions across exports	-	-	10,121

**Table note:** Shared BioSample and assembly accession counts were calculated across the provided database exports. The absence of analysis-ready overlap refers specifically to records remaining after the prespecified NCBI genotype–AST concordance and BV-BRC phenotype-linked eligibility filters. It does not establish that corresponding isolates are absent from either database, because overlap may be affected by export timing, metadata completeness, accession linkage, phenotype availability, and evidence-layer structure.

**Table 6:** Descriptive BV-BRC AMR phenotype-related coverage for commonly represented antibiotics in the *E. coli* phenotype-linked subset.

Antibiotic	Antibiotic Class	R/S/I Phenotype-Related Entries	Unique Genomes	Resistant (n)	Susceptible (n)	Intermediate (n)	Resistant Among Submitted Phenotype-Related Entries (%)
Ceftazidime	Cephalosporin/β-lactam	8010	4716	997	6951	62	12.45
Ampicillin	Penicillin/β-lactam	8010	4716	3695	4312	3	46.13
Ciprofloxacin	Fluoroquinolone	8009	4716	1374	6572	63	17.16
Trimethoprim–sulfamethoxazole	Folate-pathway inhibitor	7783	4493	2465	5280	38	31.67
Meropenem	Carbapenem/β-lactam	7835	4716	186	7647	2	2.37
Gentamicin	Aminoglycoside	7787	4493	696	6993	98	8.94
Cefotaxime	Cephalosporin/β-lactam	7704	4716	1005	6668	31	13.05
Piperacillin-tazobactam	β-lactam/β-lactamase inhibitor	7686	4493	375	7214	97	4.88
Cefuroxime	Cephalosporin/β-lactam	7198	4493	1101	6086	11	15.30
Nalidixic acid	Quinolone	5516	4716	1502	4014	0	27.23
Aztreonam	Monobactam/β-lactam	5189	4716	736	4444	9	14.18
Tobramycin	Aminoglycoside	4912	4716	580	4328	4	11.81
Cefepime	Cephalosporin/β-lactam	4814	4716	494	4305	15	10.26
Cefazolin	Cephalosporin/β-lactam	4813	4716	1168	3627	18	24.27
Ceftriaxone	Cephalosporin/β-lactam	4813	4716	946	3867	0	19.66

**Note:** This table summarizes descriptive BV-BRC phenotype-related coverage for commonly represented antibiotics among records labeled as *E. coli*. Because the export contained both Laboratory Method and Computational Method records, these values are presented only to characterize antibiotic representation and metadata structure and should not be interpreted as laboratory resistance rates, population-prevalence estimates, or external validation results. A genome could contribute more than one phenotype-related record for an antibiotic when multiple evidence entries or methods are available; therefore, the number of entries may exceed the number of unique genomes.

## Data Availability

The original contributions presented in this study are included in the article/Supplementary Materials. Further inquiries can be directed to the corresponding author. The Supplementary Materials comprise two database-specific source-data packages: the NCBI Pathogen Detection source-data package for genotype–phenotype concordance analysis, described in Appendix A.1, and the BV-BRC source-data package for metadata and phenotype-related coverage analysis, described in Appendix A.2. The post hoc amoxicillin–clavulanic acid sensitivity-analysis workbook, described in Appendix A.1, is included within the NCBI Pathogen Detection source-data package. It contains the row-level classification audit, primary and alternative confusion-matrix and performance summaries, 10,000-replicate bootstrap outputs, and computational-reproducibility information.

## Supplementary Materials

The following supporting information can be downloaded at: https://www.mdpi.com/article/10.3390/ijms27167157/s1.

## Abbreviations

AMR	Antimicrobial resistance
AST	Antimicrobial susceptibility testing
BV-BRC	Bacterial and Viral Bioinformatics Resource Center
CLSI	Clinical and Laboratory Standards Institute
ESBL	Extended-spectrum β-lactamase
EUCAST	European Committee on Antimicrobial Susceptibility Testing
FN	False negative
FP	False positive
NCBI	National Center for Biotechnology Information
NPV	Negative predictive value
PPV	Positive predictive value
POINT	Point-mutation annotation tag used in the exported NCBI Pathogen Detection AMR genotype field
TN	True negative
TP	True positive
WGS	Whole-genome sequencing

## Appendix A. Source Data Packages and Supporting Files

To support transparency and reproducibility, this study provides database-specific supplementary CSV packages derived from the exported NCBI Pathogen Detection and BV-BRC datasets. The supplementary source-data packages include the processed input tables, predefined mapping rules, and derived analytical outputs. These materials contain cleaned and harmonized analysis files and do not include raw sequencing reads or independently reprocessed genome assemblies.

### Appendix A.1. NCBI Pathogen Detection Source Data Package for Genotype–Phenotype Concordance Analysis

This package contains the processed NCBI Pathogen Detection records used for the primary genotype–phenotype concordance analysis. Files include:

(i) the filtered *E. coli*/*Shigella* organism-group dataset;
(ii) the isolate–antibiotic long-format resistant/susceptible phenotype table;
(iii) antibiotic-name harmonization outputs;
(iv) genotype-derived resistance classifications generated from exported NCBI AMR genotype annotations using predefined antibiotic-specific mapping rules;
(v) antibiotic-level TP, TN, FP, and FN classifications;
(vi) performance metrics, including sensitivity, specificity, PPV, NPV, accuracy, balanced accuracy, F1-score, and confidence intervals;
(vii) the genotype-to-antibiotic mapping framework used in Supplementary Table S3;
(viii) post hoc amoxicillin–clavulanic acid sensitivity-analysis workbook, containing the row-level classification audit, primary and alternative confusion-matrix and performance summaries, 10,000-replicate bootstrap outputs, and computational-reproducibility information.

### Appendix A.2. BV-BRC Metadata and Phenotype-Related Coverage Source-Data Package

This package contains taxonomy-strict BV-BRC genome metadata and descriptive AMR phenotype-related coverage outputs. Files include:

(i) the taxonomy-strict *E. coli*/*Shigella* genome metadata cohort;
(ii) phenotype-linked genome identifiers;
(iii) antibiotic-level phenotype-related coverage summaries;
(iv) Laboratory Method-only resistant/susceptible/intermediate summaries;
(v) Computational Method phenotype-prediction summaries;
(vi) BioSample and assembly accession overlap outputs;
(vii) descriptive AMR determinant and functional-context resources.

BV-BRC phenotype-related data were included only to document antibiotic representation, evidence-layer structure, accession overlap, and database complementarity. They were not part of the primary NCBI genotype–phenotype concordance analysis.
