# Supplementary material for: Antibiotic-Specific Genotype–Phenotype Concordance and Cross-Database Interoperability in Public Escherichia coli/Shigella WGS-AMR Metadata
Source: Int J Mol Sci. 2026 Aug 10;27(16):7157. doi: 10.3390/ijms27167157 (PMC13513162; doi:10.3390/ijms27167157)
Supplement: Supplementary file 1 [file ijms-27-07157-s001.zip › Supplementary_V5.pdf]

Supplementary Materials:

Antibiotic-Specific Genotype–Phenotype Concordance and Cross-Database Interoperability in Public *Escherichia coli*/*Shigella* WGS-AMR Metadata

**Table S1**	Public database exports, metadata fields, evidence layers, and analytical roles used in the NCBI Pathogen Detection and BV-BRC analyses.
**Table S2**	Antibiotic-level confusion matrices and extended genotype–phenotype classification performance metrics.
**Table S3**	Antibiotic-specific genotype-to-phenotype mapping rules used for NCBI AMR genotype-field interpretation.
**Table S4**	β-Lactamase determinant-family and exact-marker distributions among amoxicillin–clavulanic acid genotype-positive comparisons, stratified by phenotype agreement.
**Table S5**	Post hoc sensitivity analysis of amoxicillin–clavulanic acid genotype–phenotype performance using an alternative mapping rule in which $bla_{\mathrm{TEM\text{-}1}}$ alone was not sufficient for genotype-positive classification.

Supplementary Table S1. Public database exports, metadata fields, evidence layers, and analytical roles used in the NCBI Pathogen Detection and BV-BRC analyses.

**Variable / field**<br>**(AMRFinderPlus version)**	**Database source**<br>**(NCBI Pathogen Detection)**	**Description**<br>**(Version recorded in the exported NCBI metadata)**	**Data type**<br>**(Text/version)**	**Used in analysis**<br>**(Reproducibility and annotation provenance)**	**Harmonization/interpretation rule**<br>**(Version 4.2.7 was reported for all 10,201 final eligible records)**
**Data source**	NCBI Pathogen Detection; BV-BRC	Public database from which the record or genome was obtained	Categorical	Screening and cross-database comparison	Standardized as "NCBI Pathogen Detection" or "BV-BRC"
**Record ID / Genome ID**	NCBI Pathogen Detection; BV-BRC	Unique record or genome identifier in the exported database table	Text	Record tracking	Used to retain record-level traceability within each database
**Organism name**	NCBI Pathogen Detection; BV-BRC	Organism or taxonomic label reported in the public metadata export	Text	Inclusion filtering	Records were retained if assigned to the *Escherichia coli/Shigella* organism group
**Organism group**	Derived	Standardized organism grouping used for analysis	Categorical	Inclusion filtering	Harmonized as *E. coli/Shigella* for the primary study population
**Assembly accession**	NCBI Pathogen Detection; BV-BRC	Public genome assembly accession linked to the record or genome	Text	Inclusion filtering and cross-database overlap	Used to identify assembly-linked records and evaluate accession overlap between databases
**BioSample accession**	NCBI Pathogen Detection; BV-BRC	BioSample identifier linked to the genome or record	Text	Cross-database overlap	Used with assembly accession to evaluate overlap between NCBI Pathogen Detection and BV-BRC
**AMR genotype field**	NCBI Pathogen Detection	Exported AMR genotype annotation field containing resistance determinants	Text/list	Genotype-derived resistance classification	Parsed into determinant-level entries before antibiotic-specific mapping
**AMR determinant**	Derived from the NCBI AMR genotype field	Individual resistance gene, allele, mutation, or determinant parsed from the AMR genotype field	Text	Genotype-to-antibiotic mapping	Gene names and determinant labels were standardized where possible

**Antibiotic name**	NCBI Pathogen Detection; BV-BRC	Antibiotic reported in the AST or AMR phenotype field	Text	Antibiotic-level analysis	Harmonized to standard antibiotic names to remove spelling, capitalization, and formatting variation
**Standardized antibiotic name**	Derived	Standardized antibiotic label used in the final analysis tables	Categorical	Antibiotic-level analysis	Examples include ciprofloxacin, ampicillin, gentamicin, tetracycline, trimethoprim–sulfamethoxazole, ceftriaxone, amoxicillin–clavulanic acid, and meropenem
**Antibiotic class**	Derived	Antimicrobial class assigned to each standardized antibiotic	Categorical	Class-level interpretation	Classes included fluoroquinolone, penicillin/β-lactam, aminoglycoside, tetracycline, folate-pathway inhibitor, cephalosporin/β-lactam, β-lactam/β-lactamase inhibitor, and carbapenem/β-lactam
**AST phenotype field**	NCBI Pathogen Detection	Antimicrobial susceptibility testing phenotype summary available in the public metadata export	Text/list	Primary genotype–phenotype concordance analysis	Parsed into isolate–antibiotic phenotype entries
**AMR phenotype field**	BV-BRC	AMR phenotype information reported in the BV-BRC genome-linked phenotype tables	Text/list	Descriptive BV-BRC phenotype-related coverage analysis	Used for descriptive phenotype-related coverage and antibiotic-representation analyses
**Phenotype category**	NCBI Pathogen Detection; BV-BRC	Reported susceptibility interpretation	Categorical	Phenotype filtering and summary	Standardized as resistant, susceptible, or intermediate when available
**Resistant/susceptible phenotype status**	Derived	Binary phenotype category used for primary concordance analysis	Binary	Genotype–phenotype concordance	Resistant and susceptible entries were retained; intermediate, missing, unavailable, and non-interpretable entries were excluded from the primary R/S analysis
**R/S/I phenotype status**	BV-BRC AMR phenotype-related export	Resistant, susceptible, or intermediate phenotype category	Categorical	Descriptive BV-BRC coverage summary	May include heterogeneous evidence layers. Used only for descriptive database-coverage reporting; not interpreted as laboratory AST evidence unless

		reported in BV-BRC records			restricted to Laboratory Method records.
**Genotype-positive classification**	Derived from the NCBI AMR genotype field	Indicates that at least one mapped resistance determinant was detected for the antibiotic or antibiotic class	Binary	Concordance analysis	Assigned using predefined antibiotic-specific genotype-to-phenotype mapping rules
**Genotype-negative classification**	Derived from the NCBI AMR genotype field	Indicates that no mapped resistance determinant was detected for the antibiotic or antibiotic class	Binary	Concordance analysis	Interpreted as no mapped genomic evidence of resistance in the exported genotype field, not definitive biological susceptibility
**Concordance category**	Derived	Evidence-weighted genotype–phenotype outcome category	Categorical	Main outcome analysis	Categories were concordant resistant/true positive, concordant susceptible/true negative, genotype-positive but phenotype-susceptible / false positive, and phenotype-resistant but genotype-negative / false negative
**True positive**	Derived	Genotype-positive and AST phenotype resistant	Binary / count	Performance metrics	Used to calculate sensitivity, PPV, accuracy, F1-score, and total concordant resistant calls
**True negative**	Derived	Genotype-negative and AST phenotype susceptible	Binary / count	Performance metrics	Used to calculate specificity, NPV, accuracy, and total concordant susceptible calls
**False positive**	Derived	Genotype-positive but AST phenotype susceptible	Binary / count	Discordance analysis	Indicates genotype-positive/phenotype-susceptible discordance
**False negative**	Derived	Genotype-negative but AST phenotype resistant	Binary / count	Discordance analysis	Indicates phenotype-resistant/genotype-negative discordance
**Sensitivity**	Derived	Proportion of phenotype-resistant comparisons correctly classified as genotype-positive	Numeric percentage	Performance analysis	Calculated as TP / (TP + FN)
**Specificity**	Derived	Proportion of phenotype-susceptible comparisons	Numeric percentage	Performance analysis	Calculated as TN / (TN + FP)

		correctly classified as genotype-negative			
**Positive predictive value**	Derived	Proportion of genotype-positive comparisons that were phenotype-resistant	Numeric percentage	Performance analysis	Calculated as TP / (TP + FP)
**Negative predictive value**	Derived	Proportion of genotype-negative comparisons that were phenotype-susceptible	Numeric percentage	Performance analysis	Calculated as TN / (TN + FN)
**Accuracy**	Derived	Overall proportion of correctly classified genotype–phenotype comparisons	Numeric percentage	Performance analysis	Calculated as (TP + TN) / (TP + TN + FP + FN)
**Balanced accuracy**	Derived	Mean of sensitivity and specificity	Numeric percentage	Performance analysis	Used to reduce overinterpretation when phenotype classes are imbalanced
**F1-score**	Derived	Harmonic mean of sensitivity and positive predictive value	Numeric percentage	Performance analysis	Calculated as 2 × TP / (2 × TP + FP + FN)
**Resistant AST count**	Derived	Number of resistant AST phenotype entries for a specific antibiotic	Integer	Supplementary performance table	Calculated as TP + FN
**Susceptible AST count**	Derived	Number of susceptible AST phenotype entries for a specific antibiotic	Integer	Supplementary performance table	Calculated as TN + FP
**R/S AST comparisons**	Derived	Total resistant/susceptible isolate–antibiotic comparisons	Integer	Antibiotic inclusion and performance analysis	Calculated as resistant AST count + susceptible AST count
**R/S/I phenotype-related entries**	BV-BRC AMR phenotype-related export	Total resistant, susceptible, and intermediate phenotype-related	Integer	Descriptive BV-BRC coverage summary	May include Laboratory Method and Computational Method records. Not used as independent AST evidence, genotype–phenotype

		entries for an antibiotic			concordance data, or laboratory-only resistance estimates.
**Unique genomes**	BV-BRC	Number of distinct BV-BRC genomes with phenotype entries for a given antibiotic	Integer	Descriptive BV-BRC phenotype-related coverage analysis	Used to avoid interpreting phenotype-related rows alone as genome counts; may include genomes represented by heterogeneous BV-BRC evidence layers.
**Resistant among submitted phenotype entries (%)**	BV-BRC AMR phenotype-related export	Percentage of submitted R/S/I phenotype-related entries reported as resistant	Numeric percentage	Descriptive BV-BRC database-coverage analysis	Calculated as resistant entries divided by total R/S/I phenotype-related entries. Not interpreted as laboratory-isolate resistance, population prevalence, or clinical surveillance prevalence.
**Cross-database BioSample overlap**	Derived	Number of BioSample accessions present in both NCBI Pathogen Detection and BV-BRC exports	Integer	Cross-database comparison	Used to evaluate database overlap
**Cross-database assembly accession overlap**	Derived	Number of assembly accessions present in both NCBI Pathogen Detection and BV-BRC exports	Integer	Cross-database comparison	Used to evaluate assembly-level overlap
**Analysis-ready genotype–phenotype overlap**	Derived	Number of records satisfying the final analysis-specific eligibility criteria in both database resources	Integer	Cross-database comparison	Used to determine whether records with analytically compatible genotype–phenotype evidence overlapped between databases
**Missing / unavailable phenotype**	NCBI Pathogen Detection; BV-BRC	Phenotype entry that was absent, unavailable, or not interpretable	Categorical	Exclusion criteria	Excluded from primary resistant/susceptible concordance analysis
**Intermediate phenotype**	NCBI Pathogen Detection; BV-BRC	AST phenotype is categorized as intermediate	Categorical	Exclusion from primary R/S analysis; BV-BRC R/S/I summary	Excluded from the primary NCBI binary R/S concordance analysis. In BV-BRC, intermediate entries were retained only in descriptive phenotype-related coverage summaries and were not interpreted as laboratory AST

					evidence unless restricted to Laboratory Method records.
**Export date**	NCBI Pathogen Detection; BV-BRC	Date on which the public database file was downloaded or exported	Date	Reproducibility	NCBI Pathogen Detection data were exported on 11 June 2026; the BV-BRC genome and AMR phenotype tables were exported on 14 June 2026.
**Input file name**	NCBI Pathogen Detection; BV-BRC	Name of the exported raw or processed file used in the analysis	Text	Reproducibility	Provided in the analysis folder or supplementary data package when available
**Analysis subset**	Derived	Final subset of records used for a specific analysis	Categorical	Reproducibility and reporting	Examples include the NCBI concordance subset, the BV-BRC phenotype subset, and the cross-database overlap subset

Table note: This data dictionary defines variables used for screening, harmonization, genotype-derived resistance classification, genotype–phenotype concordance analysis, cross-database overlap assessment, and BV-BRC descriptive phenotype-related coverage summaries. Genotype-negative classifications indicate the absence of a mapped resistance determinant in the exported NCBI AMR genotype field and should not be interpreted as definitive biological susceptibility. The term *E. coli/Shigella* refers to the combined organism group used in the public database exports and should not be interpreted as species-specific estimates for E. coli or Shigella separately. For BV-BRC, only records labeled as Laboratory Method were treated as laboratory phenotype evidence when laboratory-only summaries were generated; Computational Method records were treated as model-derived predictions and were excluded from genotype–phenotype performance analyses.

Supplementary Table S2. Antibiotic-level confusion matrices and extended genotype–phenotype classification performance metrics.

Antibiotic	N R/S AST	Resistant AST, n	Susceptible AST, n	TP	TN	FP	FN	Sensitivity %	Specificity %	PPV %	NPV %	Accuracy %	Balanced accuracy %	F1-score %	95% CI for sensitivity	95% CI for specificity	95% CI for accuracy	95% CI for F1-score
**Ciprofloxacin**	7,615	1,330	6,285	1,294	5,555	730	36	97.3	88.4	63.9	99.4	89.9	92.8	77.2	96.4–98.2	87.6–89.2	89.3–90.6	75.5–78.7
**Ampicillin**	7,592	3,532	4,060	3,159	4,019	41	373	89.4	99.0	98.7	91.5	94.5	94.2	93.9	88.4–90.4	98.7–99.3	94.0–95.0	93.3–94.4
**Gentamicin**	7,344	1,129	6,215	1,057	6,112	103	72	93.6	98.3	91.1	98.8	97.6	96.0	92.4	92.1–94.9	98.0–98.7	97.2–97.9	91.2–93.5
**Tetracycline**	7,203	3,062	4,141	2,992	4,098	43	70	97.7	99.0	98.6	98.3	98.4	98.3	98.1	97.1–98.2	98.6–99.2	98.1–98.7	97.8–98.5
**Trimethoprim–sulfamethoxazole**	7,035	1,338	5,697	1,229	5,656	41	109	91.9	99.3	96.8	98.1	97.9	95.6	94.2	90.4–93.4	99.0–99.5	97.6–98.3	93.2–95.1
**Ceftriaxone**	6,841	1,411	5,430	1,300	5,394	36	111	92.1	99.3	97.3	98.0	97.9	95.7	94.6	90.7–93.5	99.1–99.5	97.5–98.2	93.7–95.4
**Amoxicillin–clavulanic acid**	6,283	801	5,482	663	3,964	1,518	138	82.8	72.3	30.4	96.6	73.6	77.5	44.5	80.0–85.3	71.2–73.5	72.6–74.7	42.2–46.6
**Meropenem**	6,228	70	6,158	56	6,146	12	14	80.0	99.8	82.4	99.8	99.6	89.9	81.2	70.3–88.9	99.7–99.9	99.4–99.7	73.0–87.5
**Total**	56,141	12,675	43,466	11,752	40,942	2,524	923	—	—	—	—	—	—	—	—	—	—	—

Table note: True positives were defined as genotype-positive and phenotype-resistant comparisons. True negatives were defined as comparisons with no mapped genomic evidence of resistance and phenotype-susceptible AST results. False positives were defined as genotype-positive/phenotype-susceptible comparisons. False negatives were defined as phenotype-resistant comparisons with no mapped genomic evidence of resistance. Sensitivity was calculated as TP/(TP + FN), specificity as TN/(TN + FP), PPV as TP/(TP + FP), NPV as TN/(TN + FN), accuracy as (TP + TN)/(TP + TN + FP + FN), and balanced accuracy as the mean of sensitivity and specificity. The F1-score was calculated as 2 × TP/(2 × TP + FP + FN). Confidence intervals are two-sided 95% percentile-bootstrap intervals derived from 10,000 isolate-level nonparametric bootstrap replicates. All values were generated using the prespecified antibiotic-specific genotype-to-phenotype mapping rules described in Supplementary Table S3. Primary results are unchanged; the post hoc alternative amoxicillin–clavulanic acid rule is reported in Supplementary Table S5.

Abbreviations: TP, true positive; TN, true negative; FP, false positive; FN, false negative; PPV, positive predictive value; NPV, negative predictive value; CI, confidence interval; R/S, resistant/susceptible.

Supplementary Table S3. Antibiotic-specific genotype-to-phenotype mapping rules used for NCBI AMR genotype-field interpretation.

**Antibiotic**	**Antibiotic class**	**AMR determinants/classes mapped to genotype-positive resistance classification**	**Rule type**	**Interpretation notes**
**Ciprofloxacin**	Fluoroquinolone	Plasmid-mediated quinolone resistance determinants, including *qnr* genes, *aac(6′)-Ib-cr*, *qepA*, *oqxA*/*oqxB*, and resistance-associated quinolone target mutations such as *gyrA*, *gyrB*, *parC*, or *parE*, when explicitly annotated in the exported genotype field.	Gene/mutation-based	Phenotypic resistance may require combinations of chromosomal target mutations and/or plasmid-mediated determinants; therefore, genotype-positive calls based on single determinants may not always correspond to phenotypic resistance.
**Ampicillin**	Penicillin / β-lactam	β-lactamase determinants with expected penicillin activity, including *bla*TEM, *bla*SHV, *bla*OXA-1-like, *bla*CTX-M, *AmpC*-type β-lactamases and carbapenemases, when present; intrinsic *bla*EC annotations were excluded from the ampicillin genotype-positive rule.	Gene-based	Ampicillin resistance was inferred from β-lactamase determinants expected to hydrolyze aminopenicillins. No mapped genomic evidence of resistance status indicates no mapped determinant in the exported genotype field, not definitive biological susceptibility.
**Gentamicin**	Aminoglycoside	Aminoglycoside-modifying enzymes with plausible gentamicin activity, including selected *aac*, *aph*, and *ant* determinants, and 16S rRNA methyltransferases such as *armA*, *rmt* genes, or *npmA* when present.	Gene-based	Not all aminoglycoside genes predict gentamicin resistance equally. Determinants mainly associated with streptomycin or spectinomycin alone should not be treated as gentamicin predictors unless supported by curated genotype annotation.
**Tetracycline**	Tetracycline	Tetracycline resistance determinants, including efflux genes such as *tetA*, *tetB*, *tetC*, *tetD*, *tetG*, *tetK*, *tetL*; ribosomal protection genes such as *tetM*, *tetO*, *tetQ*, *tetS*, *tetW*; and enzymatic inactivation genes such as *tetX*, when present.	Gene-based	Tetracycline resistance is frequently mediated by well-characterized acquired *tet* determinants; however, genotype-field interpretation may not capture determinant expression, gene integrity, or uncommon resistance mechanisms.
**Trimethoprim–sulfamethoxazole**	Folate-pathway inhibitor	Sulfonamide resistance genes *sul1*, *sul2*, *sul3*, *sul4*, together with trimethoprim resistance genes, including *dfrA*, *dfrB*, *dfrG*, *dfrK*, or related dihydrofolate reductase determinants.	Gene-based	Genotype-positive classification required the simultaneous detection of at least one trimethoprim-resistance determinant from the dfr group and at least one sulfonamide-resistance determinant from the sul group.
**Ceftriaxone**	Cephalosporin / β-lactam	Extended-spectrum β-lactamases such as *bla*CTX-M; ESBL-associated *bla*SHV or *bla*TEM variants when specified; AmpC-type β-lactamases such as *bla*CMY, *bla*DHA, *bla*FOX, *bla*ACC, *bla*ACT/MIR, *bla*MOX; and carbapenemases such as *bla*NDM, *bla*KPC,	Gene-based	Ceftriaxone resistance was inferred from β-lactamase families expected to compromise third-generation cephalosporins. Exact allele interpretation should follow the exported genotype annotation and curated β-lactamase class assignment.

		bla_{VIM}, bla_{IMP}, or bla_{OXA}-48-like when present.		
**Amoxicillin–clavulanic acid**	β-lactam/β-lactamase inhibitor	Genotype-positive classification was assigned when the exported NCBI AMR genotype field contained at least one prespecified AmpC-family, major carbapenemase, OXA-1-like, CTX-M/other ESBL, TEM-family, SHV-family, other OXA-family, or other broad acquired β-lactamase marker. Under this prespecified family-level rule, bla_{TEM-1} was included as mapped genomic evidence; therefore, bla_{TEM-1} alone was sufficient to classify an isolate–antibiotic comparison as genotype-positive for amoxicillin–clavulanic acid. Exact matched markers and their phenotype-stratified frequencies are presented in Supplementary Table S4. The post hoc alternative analysis in Supplementary Table S5 reclassified bla_{TEM-1}-only comparisons as having no mapped genomic evidence of resistance.	Gene-based, lower-confidence	This family-level mapping was considered a lower-confidence genotype-to-phenotype rule because the activity of amoxicillin–clavulanic acid may depend on exact allele identity, enzyme expression, gene dosage, inhibitor susceptibility, permeability, and co-occurring mechanisms. Because bla_{TEM-1} alone was classified as mapped genomic evidence, bla_{TEM-1}-only comparisons contributed to the genotype-positive category and could increase the statistical false-positive count. This study-specific classification does not establish phenotypic resistance or clinical actionability. AmpC-family gene detection was not interpreted as evidence of AmpC overproduction. A post hoc sensitivity analysis using a more restrictive rule is reported in Supplementary Table S5. The alternative rule is exploratory and does not establish clinical actionability or an optimal genotype-to-phenotype mapping framework. .
**Meropenem**	Carbapenem / β-lactam	Carbapenemase determinants, including bla_{NDM}, bla_{KPC}, bla_{VIM}, bla_{IMP}, bla_{OXA}-48-like, and other annotated carbapenemase families when present.	Gene-based	Meropenem genotype-positive classification was primarily based on carbapenemase detection. Non-carbapenemase mechanisms, such as porin loss combined with *ESBL* or *AmpC* production, may not be fully captured in the exported genotype field, which may lead to phenotype-resistant/no mapped genomic evidence of resistance discordance.

Table note: Genotype-positive classification was assigned when the exported genotype field contained at least one determinant mapped to the tested antibiotic under the prespecified rules reported in this table. The mapping framework was prespecified before concordance metrics were calculated and was applied uniformly across all eligible isolate–antibiotic comparisons. No antibiotic-specific rule was modified after inspecting genotype–phenotype performance. Classification as having no mapped genomic evidence of resistance indicates that no determinant was mapped to the tested antibiotic under the prespecified rule and should not be interpreted as definitive biological susceptibility. For amoxicillin–clavulanic acid, the prespecified rule included bla_{TEM-1} as mapped genomic evidence, and bla_{TEM-1} alone was sufficient for genotype-positive classification. This operational definition should not be interpreted as proof of phenotypic resistance. Determinant-level findings are provided in Supplementary Table S4.

Supplementary Table S4. β-Lactamase determinant-family and exact-marker distributions among amoxicillin–clavulanic acid genotype-positive comparisons, stratified by phenotype agreement.

Panel A: determinant-family summary

Determinant family	Genotype-positive/phenotype-susceptible, n (%)	Genotype-positive/phenotype-resistant, n (%)	Phenotype-resistant proportion among comparisons containing family
TEM family	1,081 (71.2%)	184 (27.8%)	14.5%
CTX-M family	462 (30.4%)	98 (14.8%)	17.5%
SHV family	44 (2.9%)	9 (1.4%)	17.0%
Other β-lactamase	21 (1.4%)	9 (1.4%)	30.0%
OXA-1	20 (1.3%)	62 (9.4%)	75.6%
AmpC family	12 (0.8%)	529 (79.8%)	97.8%
Other OXA family	4 (0.3%)	6 (0.9%)	60.0%
OXA-48-like carbapenemase	0	4 (0.6%)	100%
Other major carbapenemase	0	26 (3.9%)	100%

Note: Determinant-family categories were non-mutually exclusive because one comparison could contain multiple matched β-lactamase markers; therefore, percentages should not be summed. The phenotype-resistant proportion represents a descriptive proportion among genotype-positive comparisons containing the indicated family and should not be interpreted as an independently adjusted determinant-specific predictive value. AmpC-family annotation indicates gene detection only and does not demonstrate AmpC overproduction. The determinant-family results reflect the prespecified mapping framework and should not be interpreted as independently validated causal effects of individual gene families.

Panel B: Exact β-lactamase marker distribution

Exact marker	FP, n (%)	FP marker alone, n	TP, n (%)	TP marker alone, n
bla$_{TEM-1}$	1,065 (70.2%)	949	175 (26.4%)	37
bla$_{CTX-M-15}$	184 (12.1%)	107	76 (11.5%)	1
bla$_{CTX-M-27}$	140 (9.2%)	118	6 (0.9%)	1
bla$_{CTX-M-1}$	73 (4.8%)	70	4 (0.6%)	1
bla$_{CTX-M-55}$	36 (2.4%)	20	3 (0.5%)	0
bla$_{SHV-1}$	24 (1.6%)	24	0	0
bla$_{CTX-M-14}$	21 (1.4%)	13	8 (1.2%)	1
bla$_{CARB-2}$	21 (1.4%)	18	6 (0.9%)	1
Generic *bla*$_{TEM}$ annotation	21 (1.4%)	6	8 (1.2%)	1
bla$_{OXA-1}$	20 (1.3%)	2	62 (9.4%)	2
bla$_{SHV-2A}$	16 (1.1%)	15	4 (0.6%)	1
bla$_{CMY-2}$	10 (0.7%)	10	490 (73.9%)	382

Note: FP denotes genotype-positive/phenotype-susceptible comparisons, and TP denotes genotype-positive/phenotype-resistant comparisons. Marker-alone counts indicate that no other determinant included in the prespecified amoxicillin–clavulanic acid mapping rule was detected in that comparison. For the *bla*$_{TEM-1}$ row, the operational marker-alone count additionally required the absence of an exported *bla*$_{TEM}$ promoter mutation annotated as POINT. Percentages in the FP and TP columns were calculated using 1,518 genotype-positive/phenotype-susceptible comparisons and 663 genotype-positive/phenotype-resistant comparisons, respectively. Under the prespecified mapping framework described in Supplementary Table S3, *bla*$_{TEM-1}$ alone was sufficient for genotype-positive classification. Accordingly, *bla*$_{TEM-1}$ was the sole matched marker in 949 of the 1,518 genotype-positive/phenotype-susceptible comparisons (62.5%). This operational mapping definition represents study-specific genomic evidence and should not be interpreted as proof of phenotypic resistance or clinical actionability. The 949 false-positive and 37 true-positive comparisons in which *bla*$_{TEM-1}$ was the sole mapped marker were eligible for phenotype-independent reclassification under the post hoc alternative rule reported in Supplementary Table S5; no observations were removed.

Supplementary Table S5. Post hoc sensitivity analysis of amoxicillin–clavulanic acid genotype–phenotype performance using an alternative mapping rule in which *bla*$_{TEM-1}$ alone was not sufficient for genotype-positive classification.

Rule	N	Resistant AST, n	Susceptible AST, n	TP	TN	FP	FN	Sensitivity, % (95% CI)	Specificity, % (95% CI)	PPV, % (95% CI)	NPV, % (95% CI)	Accuracy, % (95% CI)	Balanced accuracy, % (95% CI)	F1-score, % (95% CI)
Primary prespecified rule	6,283	801	5,482	663	3,964	1,518	138	82.8 (80.0–85.3)	72.3 (71.2–73.5)	30.4 (28.5–32.4)	96.6 (96.1–97.2)	73.6 (72.6–74.7)	77.5 (76.1–78.9)	44.5 (42.2–46.6)
Post hoc alternative rule	6,283	801	5,482	626	4,913	569	175	78.2 (75.2–81.0)	89.6 (88.8–90.4)	52.4 (49.6–55.3)	96.6 (96.0–97.1)	88.2 (87.4–89.0)	83.9 (82.4–85.4)	62.7 (60.2–65.2)

Note: Under the primary prespecified rule, *bla*$_{TEM-1}$ alone was sufficient for genotype-positive classification. Here, "*bla*$_{TEM-1}$-only" was operationally defined as *bla*$_{TEM-1}$ being the only mapped acquired β-lactamase marker and the exported genotype field containing no *bla*$_{TEM}$ promoter mutation annotated as POINT. The post hoc alternative rule reclassified *bla*$_{TEM-1}$-only comparisons as having no mapped genomic evidence of resistance; comparisons containing *bla*$_{TEM-1}$ together with another mapped determinant remained genotype-positive, and no observations were removed. The alternative rule was applied independently of phenotype. Alternative-rule confidence intervals are two-sided 95% percentile intervals from 10,000 isolate-level nonparametric bootstrap replicates using fixed random seed 20260611. Primary-rule results are retained unchanged from the prespecified analysis. **Abbreviations:** TP, true positive; TN, true negative; FP, false positive; FN, false negative; PPV, positive predictive value; NPV, negative predictive value; CI, confidence interval.
